# Human induced pluripotent stem cells for in vitro modeling of impaired mucociliary clearance in cystic fibrosis lung disease

**DOI:** 10.1186/s13287-025-04737-0

**Published:** 2025-10-21

**Authors:** Mark-Christian Klassen, Anita Balázs, Janina Zöllner, Nicole Cleve, Laurien Czichon, Laura von Schledorn, Jan Hegermann, Janna C. Nawroth, Doris Roth, Mia Mielenz, Silke Hedtfeld, Frauke Stanke, Tihomir Rubil, Fabio Ius, Danny Jonigk, John W. Hanrahan, Arjang Ruhparwar, Ruth Olmer, Marcus A. Mall, Sylvia Merkert, Ulrich Martin

**Affiliations:** 1 Leibniz Research Laboratories for Biotechnology and Artificial Organs (LEBAO), Clinic for Cardiothoracic-, Transplantation and Vasuclar Surgery, Hannover Medical School, Carl-Neuberg-Str. 1, 30625 Hannover, Germany; 2 Biomedical Research in Endstage and Obstructive Lung Disease (BREATH), German Center for Lung Research (DZL), Hannover Medical School, 30625 Hannover, Germany; 3 Department of Pediatric Respiratory Medicine, Immunology and Critical Care Medicine, Charité-Universitätsmedizin, Berlin, Augustenburger Platz 1, 13353 Berlin, Germany; 4 German Center for Lung Research (DZL), Associated Partner Site, Berlin, Germany; 5 German Center for Child and Adolescent Heath (DZKJ), Partner Site Berlin, Berlin, Germany; 6 Cluster of Excellence ImmunoPreCept, Charité - Universitätsmedizin Berlin, Berlin, Germany; 7 Institute of Functional and Applied Anatomy, Research Core Unit Electron Microscopy, Hannover Medical School, Carl-Neuberg-Str. 1, 30625 Hannover, Germany; 8 Helmholtz Pioneer Campus and Institute of Biological and Medical Imaging, Bioengineering Center, Helmholtz Zentrum München, 85764 Neuherberg, Germany; 9 Chair of Biological Imaging at the Central Institute for Translational Cancer Research (TranslaTUM), School of Medicine and Health, Technical University of Munich, 81675 Munich, Germany; 10 Comprehensive Pneumology Center Munich, German Center for Lung Research (DZL), 81675 Munich, Germany; 11 Clinic for Pediatric Pneumology, Allergology and Neonatology, Hannover Medical School, Carl-Neuberg-Str. 1, 30625 Hannover, Germany; 12 Department of Cardiothoracic-, Transplantation and Vasuclar Surgery, Hannover Medical School, Carl-Neuberg-Str. 1, 30625 Hannover, Germany; 13 Institute of Pathology, Universitätsklinikum Aachen, AöR, Pauwelsstraße 30, 52074 Aachen, Germany; 14 Department of Physiology, McGill University, 3655 Prom Sir-William-Osler, Montreal, QC H3G 1Y6 Canada

## Abstract

Severely impaired mucociliary airway function is the primary pathomechanism in Cystic Fibrosis (CF) lung disease. Despite significant advances in CF therapy, there is still a critical need for alternative, individualized treatment options, especially for patients with untreatable CFTR mutations. Although intestinal organoids and primary airway cells are widely used as preclinical models of CF, both systems exhibit limitations with regard to the proper modelling of mucociliary clearance or the availability of sufficient cell quantities. Patient-specific human induced pluripotent stem cells (hiPSCs) are a promising alternative due to their unlimited expansion potential and capacity to differentiate into airway epithelia. However, cellular inhomogeneities in iPSC-derived airway cultures complicated conventional assays that determine CFTR function such as Ussing chamber measurements, and a comprehensive demonstration of CF pathophysiology in hiPSC-derived airway models has been largely lacking. This study provides comprehensive data demonstrating very similar gene expression, (ultra)structure and CFTR function in CF iPSC-derived airway (iALI) and primary airway (pALI) cultures. Addressing current limitations, we have implemented a sensitive, straightforward, and automatable ciliary beat frequency (CBF) assay, which is largely unaffected by inhomogeneities and directly reflects disturbed mucus viscosity and mucociliary transport in CF lung disease. Electron microscopy images confirmed the disease phenotype showing a highly dense and dehydrated mucus layer on top of CF iALI cultures. Furthermore, established CFTR modulator drugs partially rescued the disease phenotype in CF iALI cultures, which validated the utility of iALI cultures as a scalable, patient-specific platform for CF research and personalized drug development.

**Supplementary Information:**

The online version contains supplementary material available at 10.1186/s13287-025-04737-0.

## Introduction

Cystic fibrosis (CF) is a rare recessive genetic disorder that affects approximately 100,000 people worldwide. It is caused by mutations of the Cystic fibrosis transmembrane conductance regulator (CFTR) gene [1–4], which encodes a cAMP-regulated chloride and bicarbonate channel protein. Mutations in the CFTR gene have been shown to affect the transepithelial ion transport in multiple organs, with CF lung disease being the primary cause of morbidity and mortality [5–14]. To date, over 700 CF-causing mutations of the CFTR gene have been identified [15–17].

In healthy individuals, the respiratory epithelium plays a critical role in the host defense against pulmonary infection. In the airways, the secreting cell types, particularly goblet cells, produce a protective layer of mucus that covers the epithelial surface and traps inhaled pathogens. Subsequent to this initial defensive barrier, mucociliary clearance (MCC) is initiated through ciliary beating, which effectively removes mucus and pathogens from the respiratory system [18].

In CF, CFTR mutations result in reduced chloride secretion and concurrent hyperabsorption of sodium by the CFTR-regulated epithelial sodium channel (ENaC). The reduction in apical ion secretion causes the dehydration of airway mucus and the subsequent increase in mucus viscosity [19–24]. This increased viscosity impairs ciliary movement and MCC, consequently leading to chronic airway infection, inflammation, and ultimately progressive loss of lung function and lung failure [11, 25–28].

The development of highly effective small-molecule drugs, known as CFTR modulators, to restore CFTR function in CF patients has profoundly changed the clinical landscape [29, 30]. The most prominent classes of CFTR modulators include corrector drugs, which correct the folding and trafficking of the mutant CFTR protein, and potentiator drugs, which enhance the activity of the CFTR protein [30–34]. Notably, the triple CFTR modulator combination (elexacaftor-tezacaftor-ivacaftor; ETI) has been shown to provide substantial clinical benefit in CF patients carrying a Phe508del CFTR allele. However, it should be noted that ETI treatment does not fully restore CFTR function and observational studies suggest that residual abnormalities persist in patients [31, 32, 35–42]. In addition, many of the patients with other mutations do not benefit from ETI. The development of novel drugs that restore CFTR function to patients carrying other so far untreatable mutations necessitates the use of improved in vitro systems that closely reflect CF lung disease including impaired MCC as the main pathomechanism. Furthermore, the identification of genetic modifiers of CF disease, which apparently lead to a wide range of mild to severe phenotypes in patients carrying the Phe508del mutation, is imperative [43]. Such genetic modifiers may represent novel therapeutic targets in CF.

To date, intestinal organoids and primary airway cells are most commonly used for these purposes. Intestinal organoids, cultivated from rectal biopsies and analyzed by forskolin-induced swelling (FIS), function as a standard for drug testing [44]. However, their utility is limited in predicting the efficacy of CF lung disease drugs or studying airway-specific genetic modifiers [45]. This limitation also stems from their lack of cilia, which prevents them from modeling mucociliary clearance (MCC), a key feature of CF lung disease. Conversely, primary airway epithelia cultured in air-liquid interface (ALI) conditions exhibit a greater degree of similarity to the pathophysiology of CF in vivo and are instrumental in the development of personalized medicine and preclinical drug development [33, 41]. However, the supply of sufficient quantities of these cells from patients remains challenging [46]. Moreover, gene editing, particularly at the clonal level, poses a continued obstacle in both systems, making the generation of isogeneic control lines impractical.

In recent years, human induced pluripotent stem cells (hiPSCs) have emerged as a novel cell source for in vitro models of CF [47–51]. HiPSCs can easily be generated from patients that carry specific CFTR variants, for instance by reprogramming of small blood samples [50, 52]. These cells are characterized by their virtually unlimited proliferation capacity and potential to differentiate into all cell types of the human body [53, 54]. In contrast to primary intestinal and airway cells, hiPSCs facilitate gene editing on a clonal level to introduce seamless correction of mutations, gene knockouts and overexpression, and integration of reporter genes. This provides tools to study the physiologic and therapeutic relevance of candidate genes or to perform high throughput screens [50, 55–59]. Although several studies have demonstrated the differentiation of hiPSCs into airway epithelial cells in organoid or ALI cultures, most differentiation protocols are relatively complex and still associated with considerably variable differentiation efficiencies [47, 49, 60–65]. While CFTR function has already been demonstrated in these cells [47–49, 65], cellular impurities frequently complicated studies that aimed to demonstrate that hiPSC-derived airway cells closely recapitulate CF lung disease comparable to primary airway epithelial cells, which are currently considered the gold standard in the field.

In this proof-of-concept study, we sought to expand upon the findings of recent investigations by undertaking a comprehensive characterization of CF patient-specific hiPSC-derived airway epithelial cells in ALI (iALI) cultures. Our objective was to demonstrate the potential of iALI cultures as a preclinical disease model and as a drug testing platform. Our results show that despite the use of a relatively simple and straightforward differentiation protocol, iALI cultures have a high degree of similarity to primary airway (pALI) cultures, including mRNA and protein expression, mucus (ultra)structure and ion channel function. Measurement of ciliary beat frequency (CBF), which directly reflects impaired mucus viscosity and ciliary transport as a major pathomechanism in CF lung disease, was applied to iALI cultures to overcome problems associated with cellular impurities due to variable differentiation efficiencies. The CF iALI cultures carrying the Phe508del CFTR mutation closely recapitulated CF lung disease in vitro, showing a severe impairment of mucociliary function and also a reduction of chloride conductance, which was partially rescued by the triple CFTR modulator combination ETI, comparable to clinical findings. By providing an unlimited supply of patient-specific cells, iALI cultures will serve as a valuable tool in CF research. Our iALI culture platform will facilitate individualized drug development and the identification of alternative therapeutic targets. It will also accelerate the development of personalized therapies and their clinical translation.

## Results

### iALI cultures derived from healthy individuals and CF patient-specific hiPSCs share characteristic features of airway epithelial cells

**Fig. 1:**
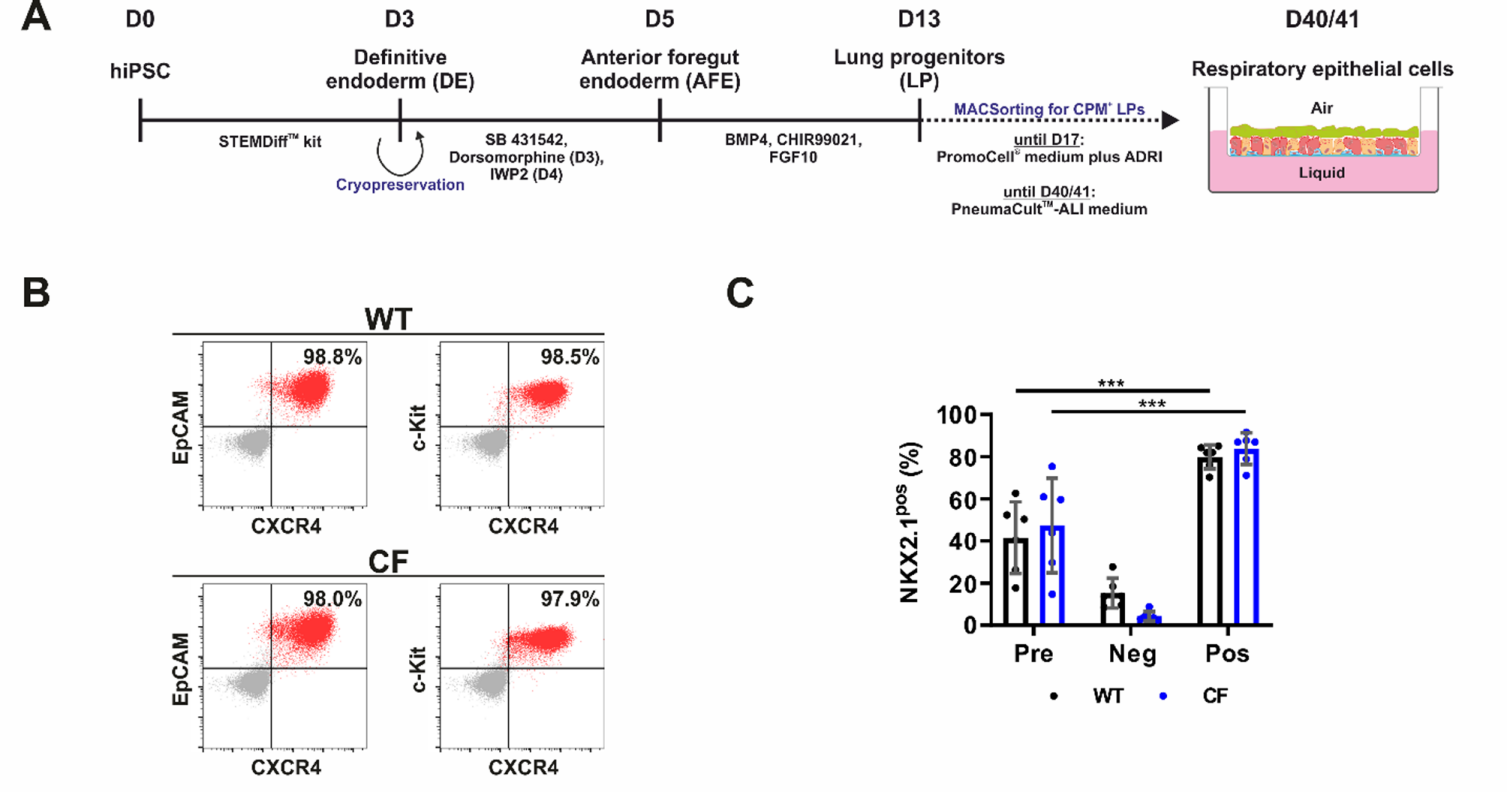
Schematic of hiPSC differentiation into iALI cultures and flow cytometry analyses of definitive endoderm and lung progenitor cell formation on day 3 and 13 of differentiation. **A** Schematic of the differentiation of hiPSCs into airway epithelial cells in air-liquid-interface culture (iALI cultures). Intermediate cryopreservation was performed at day 3 of differentiation. **B**, **C** Flow cytometry was performed to check the efficiency of definitive endoderm (DE) and lung progenitor (LP) cell formation on day 3 and 13 of differentiation, respectively. Results are shown for differentiations from WT and CF hiPSCs. **B** Flow cytometry analysis of DE generation on day 3 of differentiation (before cryopreservation). Dot blots showing expression of DE markers c-Kit, CXCR4 and EpCAM (number of differentiations: *n* = 1 per group); grey—isotype control; red—target antibody. **C** Flow cytometry analysis of NKX2.1^+^ LP cells before and after MACS on day 13 of differentiation (Pre—before sorting, Neg—negative fraction after sorting, Pos—positive fraction after sorting). Results are shown as mean ± SD, replicates represent number of differentiations: *n* = 6 per group; One-way ANOVA and post hoc Tukey test: ns—none significant, *p < 0.05, ** *p* < 0.01, *** *p* < 0.001

Healthy (wild type, WT) hiPSCs [66] and CF patient-specific (CF) hiPSCs carrying a homozygous CFTR Phe508del mutation were differentiated into iALI cultures by applying a multi-stage differentiation protocol [64, 67–69]. First, hiPSCs were differentiated into the definitive endoderm (DE) until day 3 of differentiation (Fig. 1A). Flow cytometry analysis was performed, which confirmed high DE marker expression of >97.0% in both WT and CF DE cells (Fig. 1B). As an intermediate step in the differentiation process, cryopreservation of DE cells was implemented to create WT and CF DE batches for all subsequent differentiations into iALI cultures (Fig. 1A). Following the thawing process, DE cells were differentiated into CPM^+^/NKX2.1^+^ lung progenitor (LP) cells yielding 41.6 ± 15.5% NKX2.1^+^ WT cells and 47.4 ± 20.5% NKX2.1^+^ CF cells. A magnetic-activated cell sorting (MACS) for CPM^+^ cells was performed (Fig. 1A) to enrich LP cells, yielding 79.9 ± 5.2% NKX2.1^+^ WT cells and 83.9 ± 6.8% NKX2.1^+^ CF cells (Fig. 1C). Finally, LP cells were seeded on inserts for ALI cultivation and were matured by cultivation for a minimum of an additional 27 days (Fig. 1A). To enable direct comparison with primary airway cultures as the current gold standard in CF research, we generated pALI cultures from a healthy (WT) donor and a CF patient, carrying a homozygous CFTR Phe508del mutation (CF). Our proof-of-concept study included each a single WT hiPSC line and CF hiPSC line as well as primary airway cells from a single WT and CF donor, which were all (hiPSC and primary cells) derived from independent donors.

**Fig. 2:**
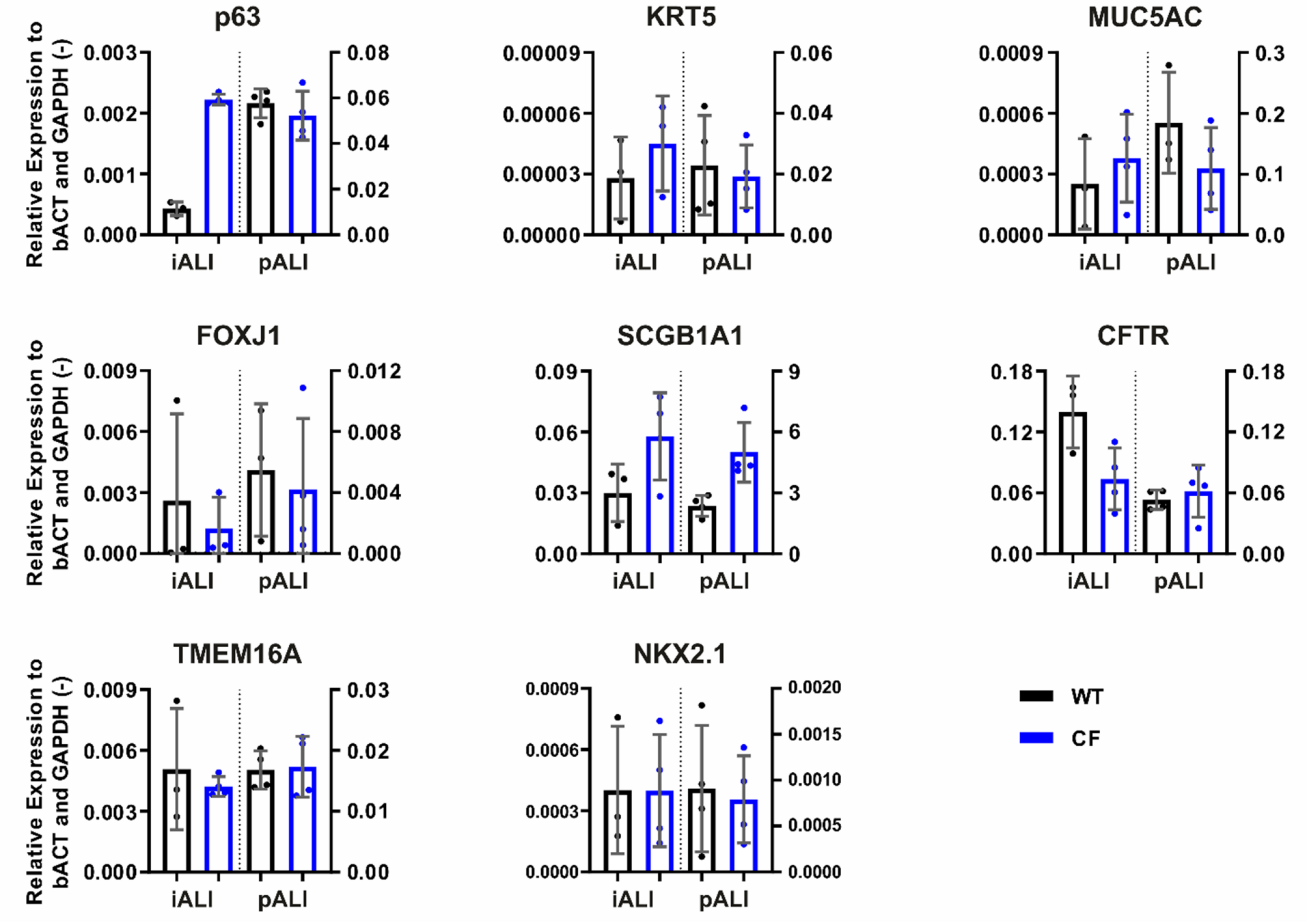
iALI cultures show transcription of mature epithelial cell type markers and epithelial ion channels. mRNA expression of airway epithelial markers for basal cells (p63, KRT5), goblet cells (MUC5AC), ciliated cells (FOXJ1), club cells (SCGB1A1) and respiratory ion channels (CFTR, TMEM16A). Results are shown as mean ± SD, replicates represent number of ALI cultures derived from independent differentiations: *n* = 3–4 per group

**Fig. 3:**
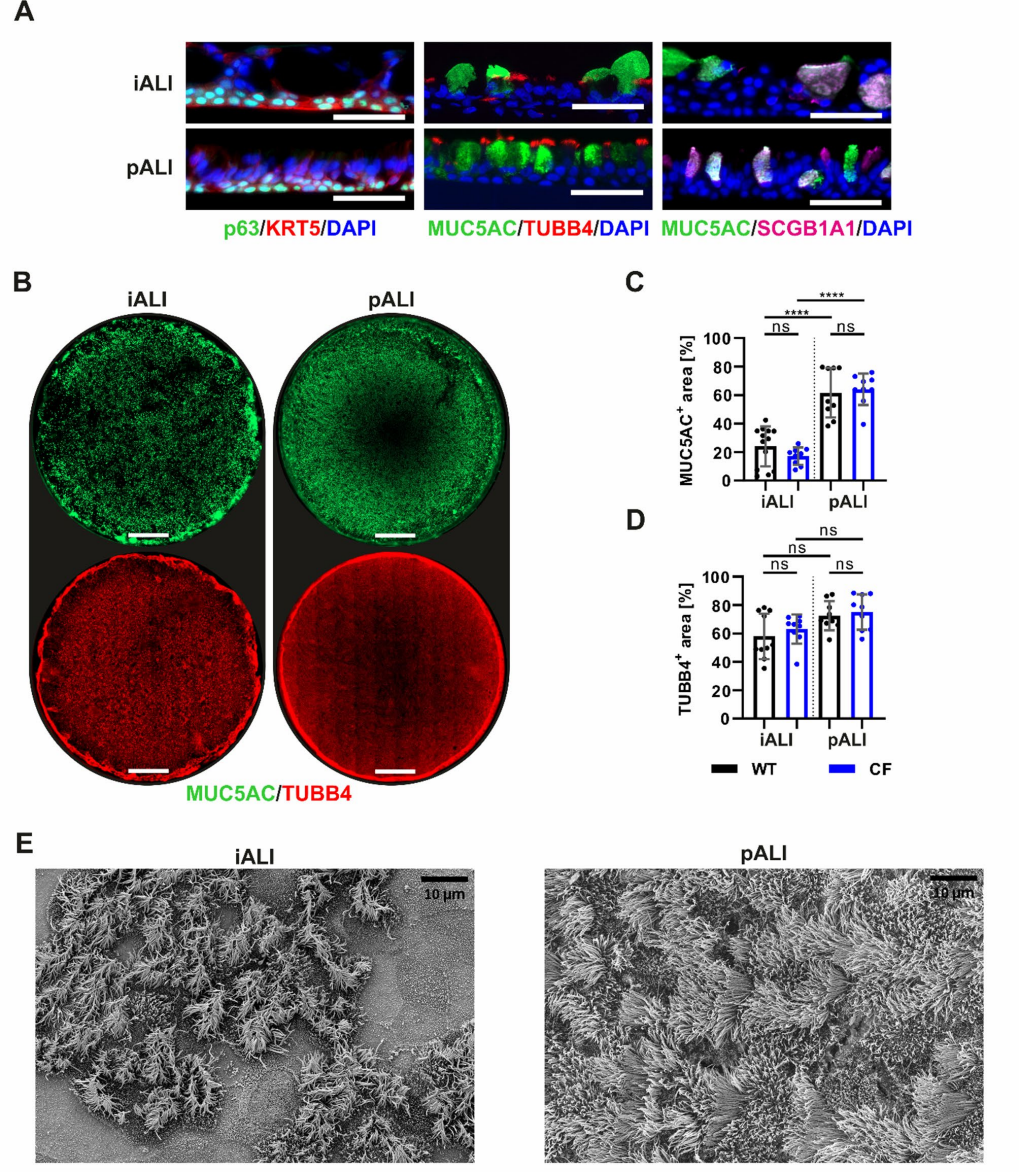
iALI cultures contain typical cellular components of mature human airway epithelium incl. a large number of ciliated cells, goblet cells and basal cells. **A** Immunofluorescence staining of paraffin sections (scale bar: 50 μm) of p63^+^/KRT5^+^ basal cells, MUC5AC^+^ goblet cells, TUBB4^+^ ciliated cells and SCGB1A1^+^ club cells. **B** Whole mount immunofluorescence staining of complete ALI insert membranes (scale bar: 2000 μm) showing the expression of MUC5AC^+^ goblet cells and TUBB4^+^ ciliated cells. Quantification of area of (**C**) MUC5AC signal and (**D**) TUBB4 signal on whole mount immunofluorescence staining of ALI insert membranes. Results are shown as mean ± SD, replicates represent number of different field-of-views of multiple ALI cultures: *n* = 9–13 per group; One-way ANOVA and post hoc Tukey test: ns—none significant, *p < 0.05, ** *p* < 0.01, *** *p* < 0.001, **** *p* < 0.0001. **E** Scanning electron microscopy (SEM) of the apical surface of ALI cultures showing multi ciliated cells and other microvilli carrying cells reflecting the more clustered distribution of ciliated cells in iALI cultures obvious also after TUBB4 staining in (**B**) (scale bar: 10 μm)

A combination of transcription analysis, immunofluorescence staining and electron microscopy was used to confirm the formation of airway epithelial cells in iALI cultures (Figs. 2 and 3, Supplemental Fig. 1). qRT-PCR analysis demonstrated the expression of epithelial cell type markers in iALI and pALI cultures including markers for basal cells (p63, KRT5), club cells (SCGB1A1), goblet cells (MUC5AC) and ciliated cells (FOXJ1) (Fig. 2). In addition, the expression of NKX2.1, a lung development marker [70], CFTR and TMEM16A, another chloride channel and a potential alternative target for CF therapies [71, 72], was detected (Fig. 2).

The prominent features and morphology of airway epithelial cells were found in both iALI cultures and pALI cultures. iALI cultures exhibited a polarized morphology (Fig. 3A) and contained p63^+^/KRT5^+^ basal cells (Fig. 3A) as well as more mature epithelial cell types, including MUC5AC^+^ goblet cells, TUBB4^+^ ciliated cells and SCGB1A1^+^ club cells (Fig. 3B-D). Additionally, iALI cultures contained BSND^+^ ionocytes, which were only found at a low frequency in pALI and iALI cultures (Supplemental Fig. 1). The immunofluorescence signal of the entire ALI insert membrane revealed comparable numbers of TUBB4^+^ ciliated cells in iALI and pALI cultures (Fig. 3D). However, the number of MUC5AC^+^ goblet cells was significantly lower in iALI cultures than in pALI cultures (Fig. 3C). Additional imaging of the apical surface via scanning electron microscopy (SEM) validated these observations and confirmed the prevalent presence of ciliated cells in iALI cultures (Fig. 3E). In accordance with the immunofluorescence staining in Fig. 3B, ciliated cells in iALI cultures appeared to be more clustered and interspersed with groups of non-ciliated cells. Morphologic differences between WT and CF epithelial cells were not observed in iALI or pALI cultures.

**Fig. 4:**
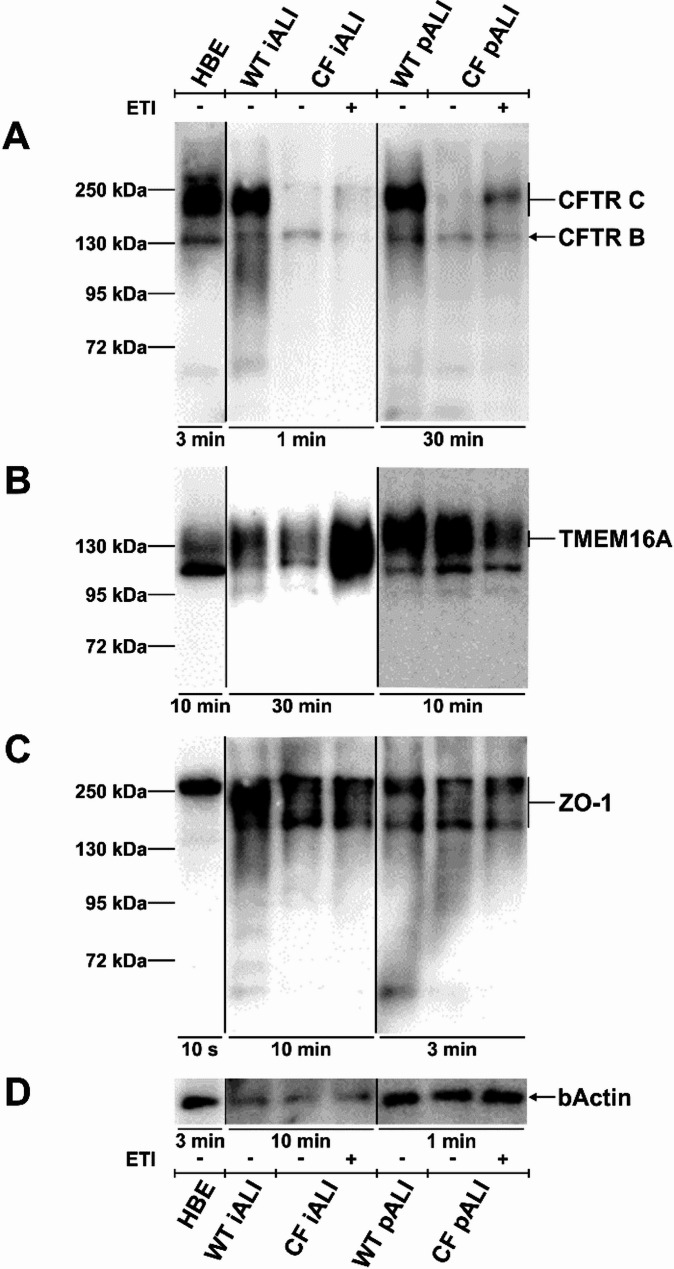
iALI cultures show protein expression of epithelial ion channels CFTR and TMEM16A as well as tight junction marker ZO-1. Western blot analyses were performed on iALI and pALI cultures as well as human bronchial epithelial cells (HBE; 16HBE14o-) as a control. Treatment with CFTR modulators ETI was applied for 48 h (WT—Healthy, CF—homozygous CFTR Phe508del, - vehicle, + ETI-treated). **A** Expression of CFTR B band at 130 kDa and CFTR C band at approx. 140–180 kDa. WT and CF^ETI^ iALI cultures expressed CFTR C band at approx. 140 kDa. **B** Staining for TMEM16A shows the expected band at approx. 120–140 kDa. **C** ZO-1 detection at approx. 195–250 kDa. **D** β-actin reference staining. All lanes were loaded with 40 µg of protein. Presented exposure times were selected for optimal display of banding pattern and are highlighted below the blot lanes. Western blot analyses have been performed as replicates of *n* = 3–4. Replicates and images of the complete blot membrane are provided in the supplementary data of Supplemental Fig. 2, Supplemental Fig. 3, Supplemental Fig. 4 and Supplemental Fig. 5

Furthermore, we performed Western blot analyses to investigate the protein expression of CFTR, of TMEM16A, a calcium-activated chloride channel [72, 73], and of the tight junction marker ZO-1, both important for proper function of the airway epithelium (Fig. 4, Supplemental Fig. 2, Supplemental Fig. 3, Supplemental Fig. 4, Supplemental Fig. 5). In both iALI and pALI cultures we detected CFTR B band at 130 kDa and CFTR C band between 140 and 180 kDa (Fig. 4A, Supplemental Fig. 2). As expected, the Phe508del mutation led to a decline in the expression of the CFTR protein, particularly the CFTR C band, in both the CF iALI and CF pALI cultures in comparison to the WT controls. Treatment with the CFTR modulator drugs ETI for 48 h (3 µM elexacaftor, 18 µM tezacaftor and 1µM ivacaftor) was applied to rescue the protein expression and function of the Phe508del mutated CFTR protein (CF^DMSO^ – vehicle, CF^ETI^ – ETI-treated). Western blot analysis revealed that the expression of CFTR C band was increased to a varying degree in CF^ETI^ iALI and CF^ETI^ pALI cultures as shown in three out of four replicates (Fig. 4A, Supplemental Fig. 2, Supplemental Fig. 5). In accordance with the qRT-PCR data, all iALI and pALI cultures showed protein expression of TMEM16A and ZO-1 (Fig. 4B + C, Supplemental Fig. 3, Supplemental Fig. 4, Supplemental Fig. 5).

### Phe508del CF iALI cultures show an impaired CFTR-dependent transepithelial chloride conductance that can be partially rescued by treatment with ETI

**Fig. 5:**
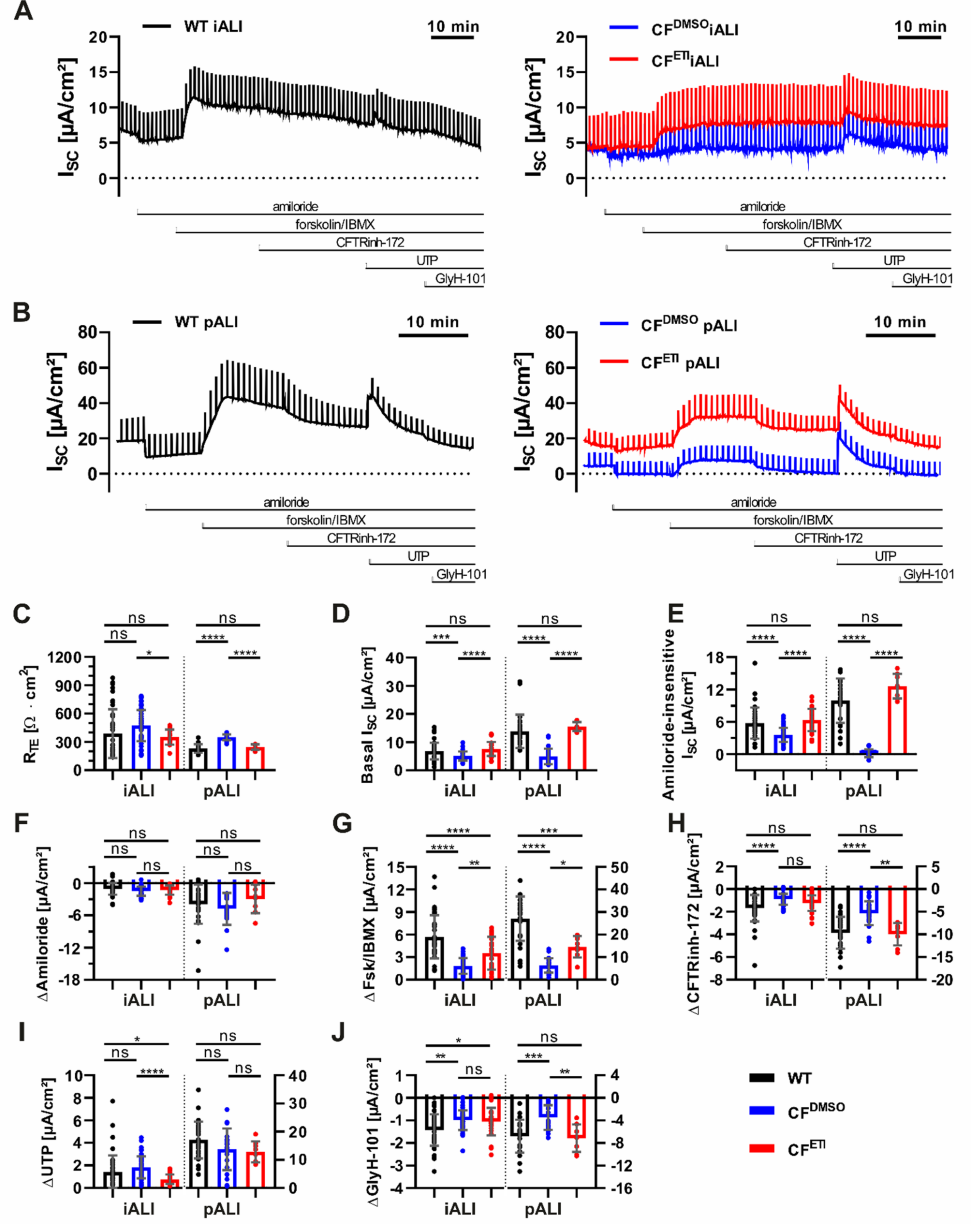
CF iALI and CF pALI cultures show an impaired transepithelial chloride transport that is partially recovered by treatment with ETI. Measurements of the transepithelial ion transport in iALI and pALI cultures by recording of the short circuit current (I_SC_) in Ussing chamber experiments. Treatment with CFTR modulators ETI was applied for 48 h (WT—Healthy, CF—homozygous CFTR Phe508del, CF^DMSO^—vehicle, CF^ETI^—ETI-treated). Exemplary original recordings of I_SC_ measurements in (**A**) iALI cultures and (**B**) pALI cultures. (**C–J**) data summary of I_SC_ measurements in iALI and pALI cultures.(**C**) transepithelial electrical resistance (R_TE_), (**D**) basal I_SC_, (**E**) amiloride-insensitive I_SC_, (**F**) amiloride-sensitive I_SC_ (ΔAmiloride), (**G**) forskolin/IBMX-sensitive I_SC_ (ΔFsk/IBMX), (**H**) CFTR inhibitor-172-sensitive I_SC_ (ΔCFTRinh-172), (**I**) UTP-sensitive I_SC_ (ΔUTP) and (**J**) GlyH-101-sensitive I_SC_ (ΔGlyH-101). Right y-axis only applies to pALI cultures if shown. Results are shown as mean ± SD, replicates represent individual ALI cultures that were derived from multiple independent differentiations: *n* = 30–57 for iALI cultures, *n* = 10–27 for pALI cultures; One-way ANOVA and post hoc Tukey test, no comparison between the iALI and pALI test group was made: ns—none significant, *p < 0.05, ** p < 0.01, *** p < 0.001, **** p < 0.0001

The bioelectric properties of WT and CF patient-derived iALI and pALI cultures were studied in Ussing chamber experiments to evaluate the basic chloride transport defect in CF iALI and CF pALI cultures, as well as to assess the rescue of mutant CFTR chloride channel function by ETI treatment (48 h) through measurements of the short circuit current (I_SC_) (Fig. 5A + B).

In both CF^DMSO^ iALI and CF^DMSO^ pALI cultures, the transepithelial electrical resistance (R_TE_) was increased (Fig. 5C), whereas the basal I_SC_ and amiloride-insensitive I_SC_ were decreased compared to respective WT ALI cultures (Fig. 5D + E). Generally pALI cultures exhibited a more pronounced amiloride response compared to iALI cultures (Fig. 5A + B + F), but no significant changes of the ΔAmiloride were observed between the WT and CF ALI cultures (Fig. 5F). This finding indicates that neither the Phe508del mutation nor ETI significantly impacted ENaC activity in the examined cultures (Fig. 5F).

Following activation of CFTR with forskolin/IBMX, both WT iALI and WT pALI cultures showed a sustained plateau phase of increased chloride transport (Fig. 5A + B + G). In accordance with the impaired CFTR-mediated chloride transport observed in individuals with CF, the recorded forskolin/IBMX-sensitive I_SC_ (ΔFsk/IBMX) was reduced in CF^DMSO^ iALI and CF^DMSO^ pALI cultures compared to their respective WT controls (Fig. 5A + B + G). iALI cultures exhibited a significant but substantially lower response after treatment with the CFTR inhibitor-172 (ΔCFTRinh-172) compared to pALI cultures. Nevertheless, the CFTR inhibitor-172 significantly reduced CFTR activity in all WT ALI cultures, and to a lesser extent in CF^DMSO^ ALI cultures, reflecting residual activity of the Phe508del mutant CFTR (Fig. 5A + B + H).

Furthermore, UTP-mediated activation of calcium activated chloride channels (CaCCs), such as TMEM16A, reached significantly greater amplitudes in pALI cultures than in iALI cultures (Fig. 5A + B + I). CF^DMSO^ iALI cultures exhibited an increased UTP-sensitive I_SC_ (ΔUTP) in comparison to CF^ETI^ iALI cultures, while no significant differences were observed between WT and CF pALI cultures (Fig. 5A + B + I).

Finally, the unspecific inhibition of chloride conductance was performed by adding of GlyH-101. As expected because of the decreased chloride transport in CFTR mutated cells, the GlyH-101-sensitive I_SC_ (ΔGlyH-101) was found to be more prominent in WT ALI cultures than in CF^DMSO^ ALI and could be observed in both cell systems (Fig. 5J).

Overall, the recorded I_SC_ confirmed the activity of epithelial ion channels, such as ENaC, CFTR and CaCCs in iALI cultures, similarly to pALI cultures. iALI cultures generally produced lower currents than pALI cultures, and measurement of the I_SC_ in iALI culture may be accompanied by a lower sensitivity for individual parameters (Fig. 5). Nonetheless, treatment with CFTR modulators ETI for 48 h resulted in a partial rescue of the impaired chloride conductance in both CF^ETI^ iALI and CF^ETI^ pALI cultures that was significant compared to untreated controls (Fig. 5). In comparison to CF^DMSO^ ALI cultures, R_TE_ was reduced in CF^ETI^ iALI and CF^ETI^ pALI cultures (Fig. 5C). Concurrently, basal I_SC_ and amiloride-insensitive I_SC_ were increased (Fig. 5D + E). Furthermore, the levels of ΔFsk/IBMX increased in both CF^ETI^ iALI and CF^ETI^ pALI cultures, accounting for approximately 62.0% and 53.9% of CFTR activity measured in WT iALI and WT pALI cultures, respectively (Fig. 5G). In addition, CF^ETI^ pALI cultures exhibited a significant increase of CFTR inhibition, as reflected by the ΔCFTRinh-172, which could not be observed in CF^ETI^ iALI cultures (Fig. 5H). Moreover, a reduction in ΔUTP was observed in CF^ETI^ iALI cultures (Fig. 5I). ΔGlyH-101 was increased in CF^ETI^ pALI cultures but not in CF^ETI^ iALI cultures (Fig. 5J).

### CF iALI cultures show altered mucus structure and mucus layer height that can be partially rescued by treatment with CFTR modulators ETI

**Fig. 6:**
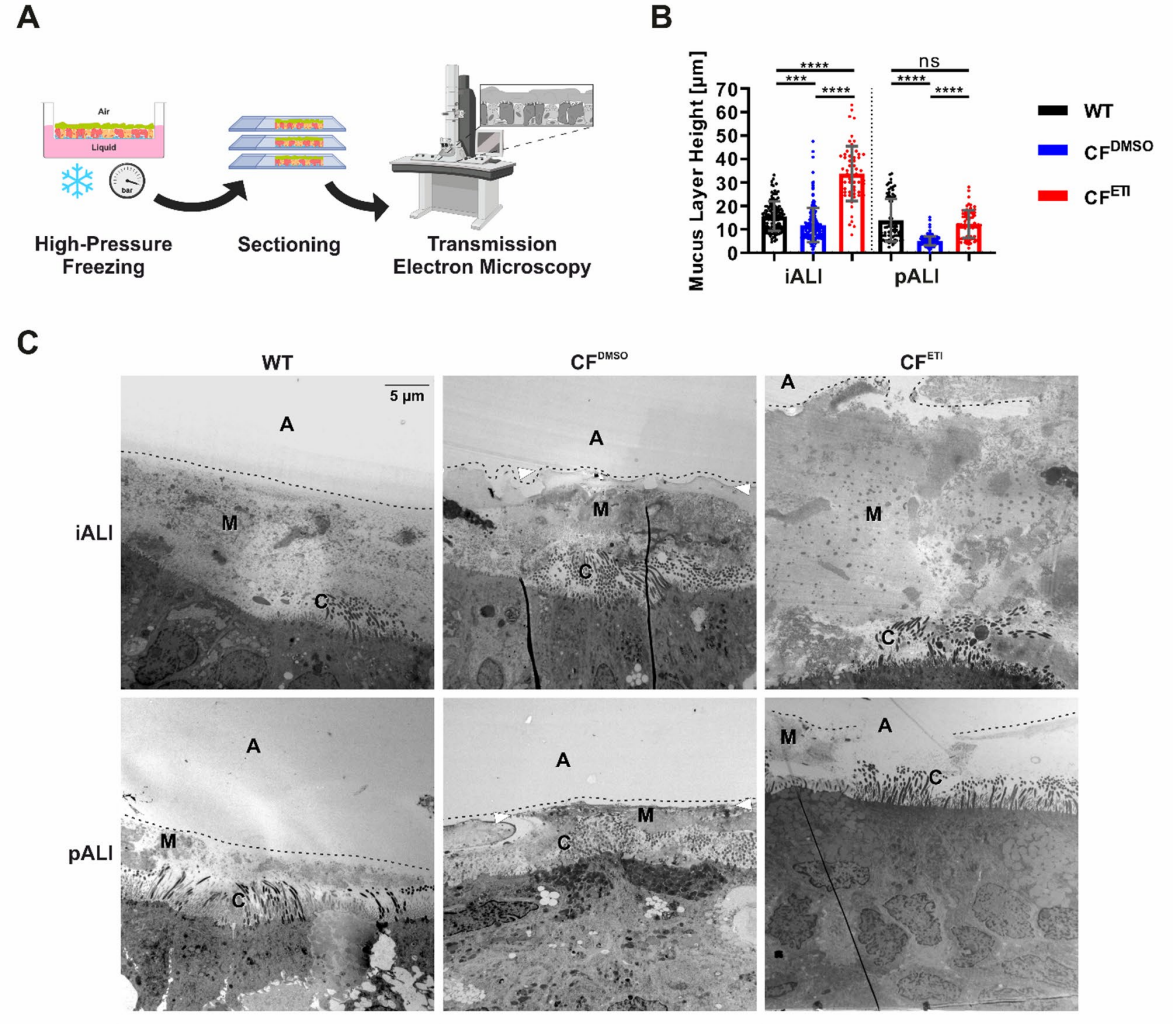
CF iALI and CF pALI cultures show a reduction of the mucus layer height compared to WT ALI cultures. **A** Sample preparation before imaging of the mucus layer by TEM (generated with Biorender). **B** Mucus layer height (MLH) measurements were manually performed on TEM images as presented in (**C**). Results are shown as mean ± SD, replicates represent technical replicates measured on single biological replicates (ALI cultures): *n* = 61–238 per group. One-way ANOVA and post hoc Tukey test, no comparison between the iALI and pALI test group was made: ns – none significant, *p < 0.05, ** p < 0.01, *** p < 0.001, **** p < 0.0001. **C** Transmission electron microscopy images of the mucus layer in iALI and pALI cultures. Results are shown at low magnification for complete overview of the cellular surface (at the bottom) and mucus layer. Structural labelling: A, air; C, cilia; M, mucus; dotted line, surface lining of the mucus layer. CF^DMSO^ iALI and CF^DMSO^ pALI cultures showed notable differences in *MLH* and quality. The mucus layer generally appeared denser and lower, with a distinctively sharp air facing surface lining (white arrowheads). On WT ALI cultures and CF^ETI^ ALI cultures, the mucus layer was higher and more dispersed, with a fissured air facing surface lining

Given that impaired mucociliary function is a hallmark of CF lung disease and appears to be at least partially rescued clinically through ETI treatment, we sought to determine whether the thickness of the mucus layer and mucus structure in CF iALI and CF pALI cultures reflect the CF disease phenotype. To this end, we characterized the ultrastructure of the mucus layer in iALI and pALI cultures from WT and CF donors by high-pressure freezing transmission electron microscopy (TEM) (Fig. 6A).

**Fig. 7:**
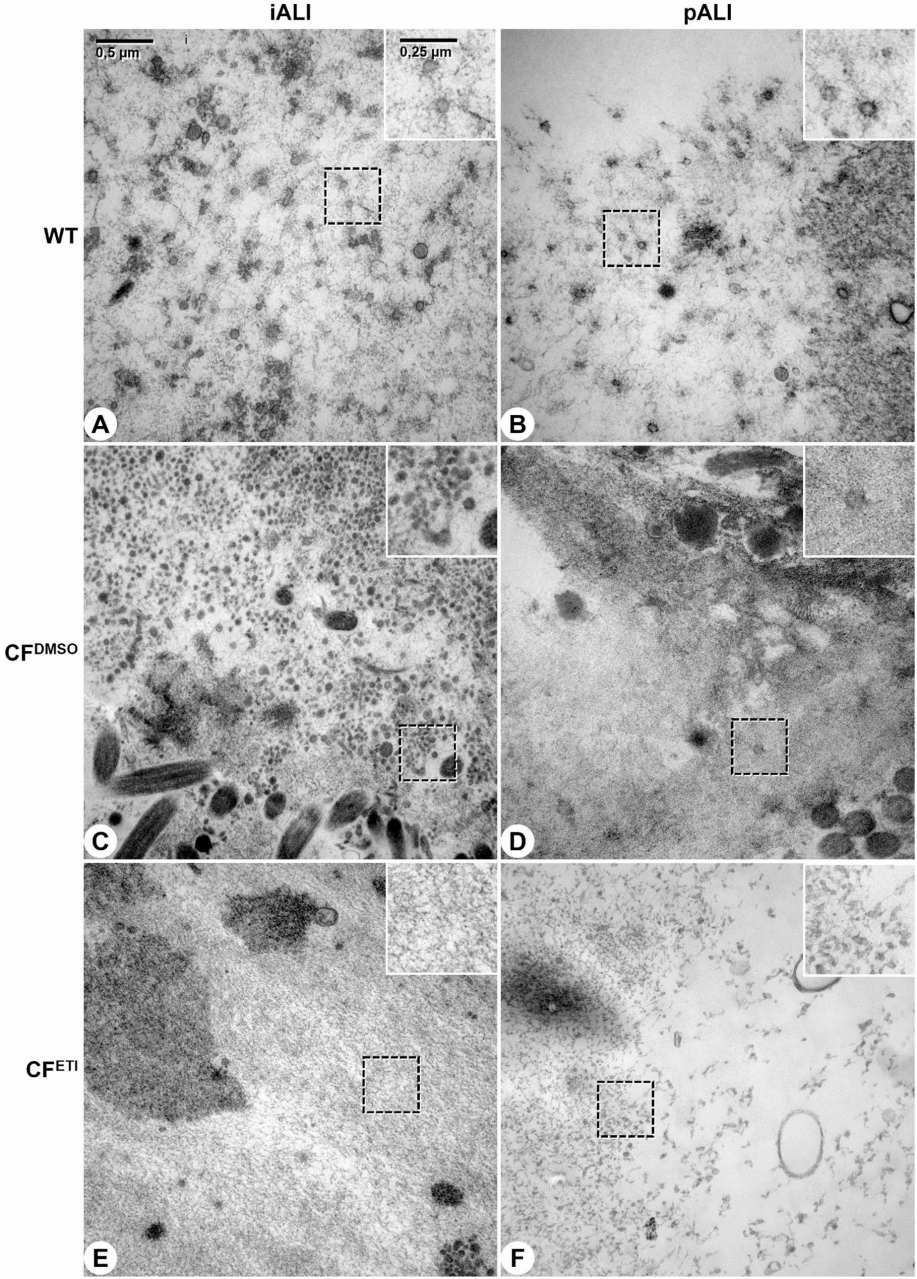
Highly dense mucus layer in CF iALI and CF pALI cultures is loosened up after treatment with CFTR modulators ETI. Transmission electron microscopy was performed on (**A + C + E**) iALI and (**B + D + F**) pALI cultures. (**A + B**): WT; (**C + D**): CF^DMSO^; (**E + F**): CF^ETI^. Treatment with CFTR modulators ETI was applied for 24 h (WT – Healthy, CF – homozygous CFTR ΔF508, CF^DMSO^ – vehicle, CF^ETI^ – ETI-treated). Results are shown at high magnification of the mucus layer. Boxed areas are depicted at doubled magnification. Scale bar shown in (**A**). (**B + E**) the mucus on CF^DMSO^ iALI and CF^DMSO^ pALI cultures showed a distinctively higher density and contain inclusions of vesicles and cellular debris. Analysis was performed on one biological replicate (ALI culture) per group (*n* = 1)

The TEM imaging and mucus height measurements revealed that the mucus layer exhibited a comparable structure and properties in iALI and pALI cultures (Figs. 6 and 7, Supplemental Fig. 6). In all ALI cultures, the mucus layer was composed of more or less densely packed molecular networks, which were visible throughout most of the prepared cross sections (Figs. 6C and 7, Supplemental Fig. 6), and exhibited a mucus layer height (MLH) of variable degree (Fig. 6B). The CF^DMSO^ ALI cultures exhibited a denser mucus layer that typically showed a distinctive surface lining, which closely reflected the in vivo phenotype of CF lung disease. In contrast, the mucus layer in WT ALI cultures was less dense and showed a loose surface lining (Figs. 6B and 7, Supplemental Fig. 6). The measured MLH was generally lower in CF^DMSO^ ALI cultures than in WT cultures in both the iALI and pALI culture systems (Fig. 6B + C). Treatment with ETI for 24 h not only increased the MLH in CF^ETI^ iALI and CF^ETI^ pALI cultures but resulted in an even thicker mucus layer in CF^ETI^ iALI cultures than in WT iALI cultures (Fig. 6B + C). In addition, the mucus structure was less dense and showed a loose surface lining in CF^ETI^ iALI and CF^ETI^ pALI cultures (Figs. 6C and 7, Supplemental Fig. 6). These findings suggest a rehydration of the mucus layer and complete rescue of the disease phenotype by ETI in the iALI culture system, which so far could not be observed or concluded in Ussing chamber measurements (Fig. 5). Furthermore, TEM imaging confirmed the presence of cilia and microvilli in both iALI and pALI cultures (Fig. 6, Supplemental Fig. 6).

### CF iALI cultures show decreased ciliary beat frequency that can be partially rescued by treatment with CFTR modulators ETI

**Fig. 8:**
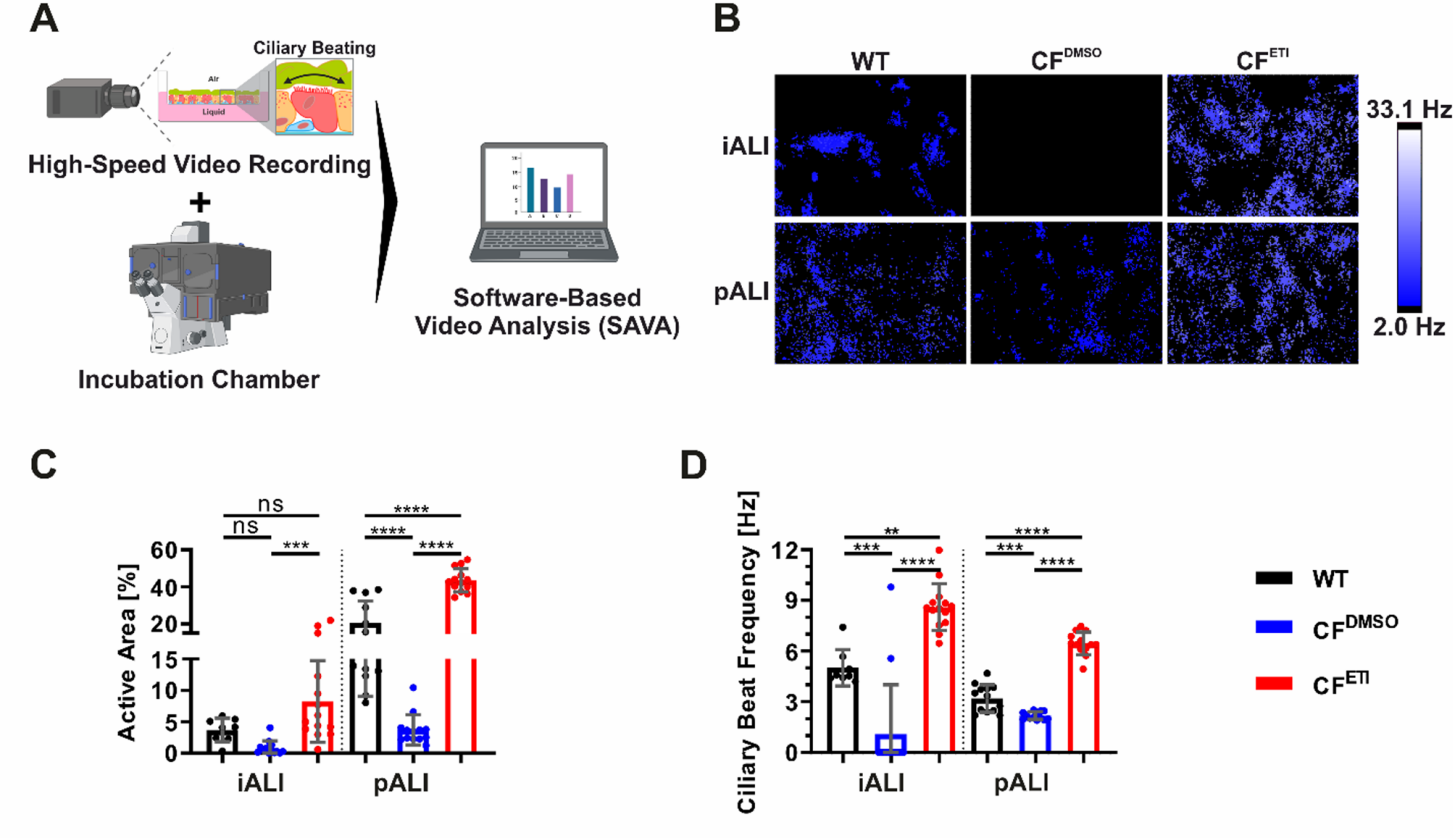
CF iALI and CF pALI cultures show an reduction of ciliary beating and corresponding impairment of mucociliary function that could be recovered by treatment with ETI. **A** Essential components for the measurement of the ciliary beat frequency (CBF) and corresponding active area of ciliary motion using high-speed video recordings and SAVA software-based analysis (generated with Biorender). Treatment with CFTR modulators ETI was applied for 24 h (WT—Healthy, CF—homozygous CFTR ΔF508, CF^DMSO^—vehicle, CF^ETI^—ETI-treated). **B** Heat maps showing exemplary fields of view of the CBF measurement. Data summary of (**C**) the active area of ciliary motion and (**D**) the CBF recorded in iALI and pALI cultures. Results are shown as mean ± SD, replicates represent individual ALI cultures that were derived from multiple independent differentiations: *n* = 8–14 per group. One-way ANOVA and post hoc Tukey test, no comparison between the iALI and pALI test group was made: ns—none significant, *p < 0.05, ** p < 0.01, *** p < 0.001, **** p < 0.0001

CFTR dysfunction leads to airway surface dehydration, which results in the secretion of highly viscoelastic mucus that impairs proper ciliary function and thus clearance of mucus, pathogens and other inhaled particles. Due to the laborious and challenging nature of MLH measurements and mucus structure visualization via TEM (Fig. 6), we sought an alternative method to directly quantify impairment of MCC, which largely determines the phenotype of CF lung disease. Given that the decreased chloride transport in CF results in elevated mucus viscosity and subsequent impairment of ciliary movement and MCC, we opted to utilize high-speed video microscopy to assess the ciliary beat frequency (CBF). Notably our approach involved the utilization of a specialized software tool (SAVA) that facilitates automated analysis (Fig. 8A). This software was configured to quantify the active areas of ciliary movement as well as the CBF.

Motile cilia were detected in all iALI and pALI cultures and measurements confirmed a generally reduced active area of ciliary beating in iALI cultures compared to pALI cultures (Fig. 8B + C). This finding corresponds with the more clustered appearance of ciliated cells (Fig. 3B + E). However, the CBF levels in WT iALI cultures generally surpassed those observed in WT pALI cultures (Fig. 8D). While CF iALI cultures measurements exhibited a less distinct phenotype in the Ussing chamber compared to CF pALI cultures (Fig. 5), CF iALI cultures demonstrated an even more pronounced disease phenotype in the CBF assay, characterized by a significant impairment of the ciliary beating (Fig. 8C + D). Despite the presence of ciliated cells, the active area and CBF were significantly diminished in CF^DMSO^ pALI cultures, and in CF^DMSO^ iALI cultures the ciliary beating was either not visible in the majority of cultures, or below the detection limit (2 Hz) of the software (Fig. 8B-D). Following 24 h of ETI treatment, a significant augmentation in the active area and CBF was observed in both CF^ETI^ iALI and CF^ETI^ pALI cultures (Fig. 8B-D). Also, all CF^ETI^ ALI cultures generally exhibited a greater CBF than the respective WT controls (Fig. 8D). Similar to the MLH measurements (Fig. 6), these findings suggest a rehydration of the mucus layer that consequently reduced the mucus viscosity and accelerated the ciliary beating (Fig. 8C + D). Again, ETI treatment led to a complete rescue of the disease phenotype and even overcompensation of the CBF in both CF^ETI^ iALI and CF^ETI^ pALI cultures (Fig. 8D).

## Discussion

In CF research, reliable patient-specific in vitro models are a crucial to developing novel CFTR modulator drug against currently untreatable CFTR mutations and identifying genetic modifiers as novel therapeutic targets. Although, intestinal organoids and primary airway cells are currently considered the gold standards in the field, both systems face particular limitations concerning either their ability to reflect important aspects of CF lung disease or the availability of sufficient quantities of patient-specific cells, respectively.

In principle, all these issues and limitations can be adequately addressed in organotypic models consisting of airway epithelial cells derived from patient-specific hiPSCs. Several studies demonstrated the differentiation of hiPSCs into airway epithelial cells. However, currently available differentiation protocols face two major difficulties. On the one hand, many of the available protocols results in highly variable outcomes in terms of efficiency and cellular purity. As a result, the sensitivity of FIS assays and electrophysiological measurements are compromised [47, 49, 64, 65]. On the other hand, more advanced protocols, namely those of Hawkins and colleagues, are difficult to establish due to their relatively high complexity and are more cost- and time-intensive than others [47, 49, 60–65], which is critical in particular on a higher throughput scale as often required in drug development.

Going beyond published work, we focused on a comprehensive comparison of organotypic hiPSC-derived airway cultures (iALI cultures) and primary airway cultures (pALI cultures), the current gold standard in CF research, in terms of proper gene and protein expression, structural characteristics and functionality. We demonstrate the usefulness of these iALI cultures, generated via a relatively simple and fast protocol that is easily scalable in 2D and does not require intermediate organoid cultivation. Application of an automatable CBF assay allows determination of decreased CBF which directly reflects reduced mucociliary clearance due to increased mucus viscosity as key pathomechanism of CF lung disease. Our results show that this assay is not affected by a variable cellular composition of iALI cultures and suggest an even higher sensitivity than measurement of pALI cultures.

We verified that iALI cultures contain a structured and polarized airway epithelium, which is composed of basal cells, ciliated cells, club cells, goblet cells and ionocytes, and which exhibit expression of CFTR and TMEM16A. In both iALI and pALI cultures, minor variations in the expression levels were observed between WT and CF cells, which however are likely not dependent on the mutated CFTR gene. It can be assumed that these variations reflect donor differences. In case of iALI cultures they may also reflect the outcome of differentiation, which depends on the donor or cell clone, and may lead to heterogeneity in the form of different cell composition and states of maturity. The low magnification images of the entire wells stained for MUC5AC and TUBB4 illustrate that iALI cultures contain a high abundance of ciliated and goblet cells often arranged as clusters. Electron microscopy revealed that in iALI cultures, in contrast to pALI cultures, regions covered with ciliated cells were interspersed with flat cells that may comprise non-ciliated epithelia as well as other cell types. Of particular note are vimentin^+^ mesenchymal cell lineages (data not shown), which represent cellular contaminants that are to a limited degree carried over during MACS of CPM^+^/NKX2.1^+^ LP cells.

While the significance of ionocytes in CF lung disease remains to be elucidated, a low number of ionocytes were also detected in iALI cultures. It is noteworthy that these ionocytes have been described as a rare epithelial cell type that exhibits exceptionally high levels of CFTR expression and might be of particular interest for CF research in the future [74, 75].

Typical for WT-CFTR, we detected a much lower expression of the immature core-glycosylated glycoisoform B (CFTR B) in both WT pALI and iALI cultures, than of the mature complex glycosylated glycoisoform C (CFTR C). According to the findings of previous studies [76, 77], our CF cultures both exhibit a substantially lower level of the CFTR C compared to the CFTR B. Notably, our data indicate an even more pronounced effect of the Phe508del mutation compared to the recent published findings [76]. Finally, although a substantial variation was observed between biological replicates for iALI and pALI cultures, ETI treatment increased the proportion of the CFTR C, which is consistent with previously published data [77, 78]. These findings provide further evidence that iALI cultures behave comparable with regard to the effect of CFTR correctors (elexcaftor and tezacaftor) on CFTR glycosylation and maturation, both crucial for trafficking and stability.

Aside from this, we identified protein expression for TMEM16A, a potential alternative target for CF therapies [6, 79], and ZO-1. While the expression levels of ZO-1 remained consistent across all samples, TMEM16A expression exhibited significant variability. This variability appeared to be independent of the presence of the Phe508del mutation and may rather be donor- or, in case of iALI cultures, clone-dependent, and may additionally be influenced by the differentiation outcome.

Overall, iALI cultures demonstrated a significant degree of molecular similarity to the epithelial cells present in pALI cultures. The Phe508del mutation resulted in reduced expression of CFTR C protein, while other variations between WT and CF cultures were likely due to cell line- and clone-specific factors. Additionally, cellular contaminants carried over during MACS of CPM^+^/NKX2.1^+^ LP cells were the most probable source of molecular differences between iALI and pALI cultures. However, these contaminants did not compromise the utility of iALI cultures for modeling CF lung disease, as discussed below.

At the functional level of the airway epithelial cells, Ussing chamber experiments have demonstrated the activity of epithelial ion channels, including ENaC, CFTR, and CaCCs, in iALI cultures. While the activity of CFTR could be confirmed by means of stimulation with forskolin/IBMX, we also assume the activity of other GlyH-101-sensitive chloride channels, such as SLC26A9 [80], in iALI and pALI cultures, as CFTR inhibitor-172 did not fully inhibit CFTR. In addition, our findings are consistent with previous reports, demonstrating that ETI only partially restores chloride transport in CF ALI cultures despite its considerable therapeutic benefit for patients with at least one Phe508del mutation [32, 36].

Nonetheless, we also detected that iALI cultures generally produced lower currents than pALI cultures. Therefore, electrophysiological measurements in iALI cultures may be accompanied by a lower sensitivity for individual parameters. It is possible that the lower degree of maturity of the epithelial cells in iALI cultures contributes to this observation. However, it is more likely that contaminating non-epithelial cells, especially vimentin^+^ mesenchymal cell lineages typically detectable at varying numbers, account for the lower currents and correspondingly lower assay sensitivity. Depending on the differentiation outcome, cells that lack expression of relevant ion channels may actually cover a considerable part of the ALI membranes between clusters of ciliated epithelia. These cells may substantially influence the measurement of I_SC_.

According to these results, we anticipated that the lower purity of our iALI cultures, compared to pALI cultures, would negatively affect the manifestation of CF-typical impairments in mucociliary function, such as reduced MLH, increased mucus density, and reduced CBF (due to dehydration and leading to increased viscosity). However, contaminating non-epithelial cells did not negatively affect MLH or mucus density, enabling accurate modeling of mucus impairment in CF lung disease. The mucus layer in iALI cultures was thicker than in pALI cultures, and the effect of the Phe508del-mutated CFTR was evident in both systems. In contrast to the measured chloride transport and CFTR activity, ETI treatment fully restored the MLH of CF ALI cultures to WT levels, even exceeding them in iALI cultures. Although not quantitatively assessed, ETI also restored the looser mucus density in CF iALI cultures, as observed in WT controls. Together, these findings indicate rehydration of the mucus layer following ETI treatment and highlight the significant clinical benefits of ETI treatment observed in CF patients [30–32, 36, 40]. Compared to electrophysiological measurements or the FIS assay in organoids, the measurement of MLH and mucus density may provide a more accurate reflection of the therapeutic effect of CF modulators on CF lung disease. Still, utilizing electron microscopy to evaluate the MLH and mucus density remains a very laborious and time-consuming process.

We further sought an alternative approach to evaluate the impairment of MCC in CF. We considered tracking of mucociliary transport by the use of fluorescent particles as not suitable due to the reduced areas covered by ciliated cells in iALI cultures [69]. Therefore, we employed the CBF assay as a significantly more useful, straightforward and time-efficient tool. In particular, the CBF assay could potentially be automated and adapted for medium- and high-throughput applications, making it a promising tool for large-scale studies of MCC impairment in CF models. While pALI cultures exhibited substantially more active areas of ciliary beating, CBF levels remained comparable in iALI and pALI cultures. In comparison to pALI cultures, the effect of the Phe508del mutation was even more pronounced in iALI cultures, suggesting a more sensitive readout in iALI cultures. Certainly, further analyses of primary cells and iPSC-derived airway cells from different patients have to confirm whether iALI cultures are generally more sensitive than primary airway cells. Nevertheless, we can confidently conclude that analysis of CBF on iPSC-derived airway cells largely eliminates the current problem of variable differentiation efficiencies and closely reflects changes in mucociliary transport in the airways caused by CFTR mutations and CFTR modulator drugs. Notably, the application of ETI resulted in a CBF that surpassed the CBF in WT iALI cultures, suggesting that this approach may more accurately reflect the substantial therapeutic efficacy of ETI than the observed partial rescue of chloride transport function as measured by electrophysiological techniques or FIS assays. To our knowledge, there are currently no reports indicating that ETI treatment results in an overcompensated recovery of mucociliary function in vivo [40, 81]. However, some in vitro studies suggest that CFTR modulator drugs can exhibit off-target effects affecting the activity of other epithelial ion channels, which may contribute to the observed overcompensation in both iALI and pALI culture systems [82, 83].

We believe that our iALI culture system, in combination with the CBF assay, offers a user-friendly platform that can be easily adopted by other laboratories and the broader CF research community. First, the relative simplicity of our differentiation protocol provides significant advantages, making our platform more time- and resource-efficient compared to existing protocols. For example, the current state-of-the-art protocol developed by Hawkins and colleagues includes a targeted basal cell generation step prior to ALI culture seeding, which significantly increases culture purity [49]. However, this protocol necessitates additional differentiation steps in Matrigel-based organoid cultures, which are both highly time- and cost-intensive even before ALI seeding. In contrast, our iALI cultures are derived from 2D cultures that require less expensive materials and can be easily scaled to produce larger quantities of cells. Second, despite the presence of considerable cellular contaminants in iALI cultures, our CBF assay remains unaffected by cellular contaminations, providing a robust readout of the primary defect in CF lung disease and demonstrating sensitivity to clinically relevant CFTR modulator drugs. Moreover, the CBF assay utilizes a straightforward setup consisting of an incubator chamber microscope equipped with a high-speed camera and the SAVA software for video recording and analysis. As mentioned before, the CBF assay could even be automated to enable screenings for novel CFTR modulator drugs. In comparison, other standard methods such as electrophysiological measurements in Ussing chambers require greater technical expertise, enable only a low sample throughput and provide no conclusive evidence for effects on the main impairment of MCC in CF lung disease.

## Conclusions

In summary, our proof-of-concept study demonstrates that patient-specific iALI cultures are indeed an excellent in vitro model of CF lung disease. We have leveraged the vast majority of the theoretical advantages of hiPSCs over immortalized cell lines, intestinal organoids, and primary airway cells. Taking advantage of the unlimited expansion potential of hiPSCs, which allows even large-scale production of individual patient-derived cells [84, 85], we demonstrated that complex organotypic cultures can be generated with characteristic (ultra)structural features of highly polarized airway epithelial cells, including ciliated cells, goblet cells, club cells and underlying basal cells, covered with mucus whose height and density are contingent on CFTR function. We have demonstrated the functionality of CFTR and other characteristic ion channels relevant for CF lung disease at the electrophysiological level, including the effect of CF modulators that closely reflect clinical data. These functional studies have been complemented by an innovative in vitro assay that is suitable for high throughput applications to determine CBF. The CBF assay in iALI cultures has been shown to more closely reflect impaired MCC as the predominant pathomechanism of CF lung disease than existing in vitro systems. Furthermore, it has the capacity to directly visualize the effects of genomic modifiers of CF, as well as of novel drugs and drug combinations, on rare CFTR mutations that have remained untreatable to date.

## Methods

###  Cell culture of hiPSCs

 A CFTR wild type hiPSC line (MHHi001-A) [66] (wild type, WT) and a hiPSC line (MHHi002-A) carrying a homozygous CFTR Phe508del mutation as a diseased reference (CF) were used for all experiments. HiPSC lines were cultured in 5–8 mL of E8 medium (self-made) on Geltrex^®^ (Thermo Fisher Science, A1413202)-coated culture vessels (25 cm² surface area). Media changes were conducted daily. Every three to four days the cells were passaged by dissociation with 1 mL Accutase™ (Sigma-Aldrich, A6964) and seeded at a density of 3.2 × 10^4^ cells cm^− 2^ while supplementing the medium with 10 µM ROCK inhibitor Y27632 (RI) (Tocris; 1254). All cells were maintained at 37 °C and 5% CO_2_.

###  Differentiation of hiPSCs into airway epithelial cells (iALI cultures)

HiPSCs were differentiated towards definitive endoderm (DE) utilizing the STEMdiff™ Definitive Endoderm Kit (STEMCELL Technologies, 05115). Seven days (day − 7) before induction of the DE differentiation, hiPSCs were passaged and seeded at a density of 1.2 × 10^4^ cells cm^− 2^ on a 25 cm² culture vessel. From day − 3 onward the 8 mL E8 medium was supplemented with STEMdiff™ Definitive Endoderm TeSR™-E8™ Supplement (1:20). On day − 1 the cells were passaged and seeded at a density of 3.33 × 10^4^ cells cm^− 2^ on a 25 cm² culture vessel. In intervals of 24 h the media was changes to 5 mL of STEMdiff™ Endoderm Basal Medium supplemented with MR (1:100) and CJ (1:100) for the first 24 h and then supplement CJ for further 48 h. On day 3, the DE cells were dissociated with 1 mL Accutase™ and seeded at a density of 1.35 × 10^5^ cells cm^− 2^ on Geltrex^®^-coated 6-well plates in 2 mL of basis medium (BM) supplemented with 3 µM Dorsomorphin (Merck, P5499), 10 µM SB431542 (kindly provided by A. Kirschning, Leibniz University Hannover) and 10 µM RI. All further media changes were conducted in intervals of 24 h. On day 4, media was changed to BM supplemented with 2 µM IWP2 (Torcis, 3533) and 10 µM SB431542. From day 5 until day 12, media changes were conducted with BM supplemented with 10 μm BMP4 (R&D Systems, 314-BP), 3 µM CHIR99021 (kindly provided by A. Kirschning, Leibniz University Hannover) and 10 nM FGF10 (R&D Systems; 345-FG). On day 13, cells were dissociated with 2 mL of Accutase™ per well and magnetic-activated cell sorting (MACS) was applied to enrich CPM^+^ lung progenitor cells. The following quantities are appropriate for MACS of 10^7^ cells. MACS was performed by resuspension of cells in 320 µL MACS buffer (selfmade), initial blocking of the cells with 80 µL FcR blocking reagents (Miltenyi Biotec, 130-059-901) for 20 min and staining with 2µL anti-CPM antibody (FUJIFILM Wako, 014-27501) for 20 min. After centrifugation, cells were resuspended in 150 µL MACS buffer and labelling with 50 µL anti-mouse IgG MicroBeads (Miltenyi Biotec, 130-047-201) was performed. Cells were centrifuged and resuspended in 3 mL of Knockout™ DMEM (Thermo Fisher Science, 10829018) before performing cell separation in LS columns (Miltenyi Biotec, 130-042-401). For further maturation in ALI culture, CPM^+^ cells were either seeded at a density of 2.65 × 10^5^ cells cm^− 2^ on ThinCert^®^ inserts (Greiner Bio-One, 665641) or Costar^®^ Snapwell™ inserts (Corning, 3801) coated with 804G medium (self-made) [86]. Cells were cultured in Small Airway Epithelial Cell Growth medium (PromoCell, C-21070) supplemented with 1.0 µM A 83 − 01 (Tocris, 2939), 0.2 µM DMH-1 (Tocris, 4126) and 5 µM RI (ADRI) for 96 h (adapted from Mou et al. 2016 and McCauley et al. 2017) [64, 86] and 48 h in in PneumaCult™-ALI medium (STEMCELL Technologies, 05001) before apical medium was removed to induce air-liquid-interface (ALI) cultures. ALI cultures were provided with 0.5 mL of medium from the apical side and 1–3 mL from the basolateral side. Media changes on the basolateral side were conducted every two to three days. Molecular and functional analyses were performed earliest on day 40/41 of differentiation. All iALI cultures were stimulated IL-13 (10 ng mL^− 1^; PeproTech, 200 − 13). In addition, CF ALI cultures were treated with elexacaftor (3 µM, Selleckchem, S8851), tezacaftor (18 µM, Selleckchem, S7059) and ivacaftor (1 µM, Selleckchem, S1144) (ETI) for 24–48 h before analysis. All cells were maintained at 37 °C and 5% CO_2_.

### Isolation, expansion and differentiation of primary human airway epithelial cells (pALI cultures)

Human explanted lung tissue was obtained from the Hannover Lung Transplant Program after patients informed written consent. The tissue samples were thoroughly cleared of excess tissue to isolated the bronchial airways, cut into small pieces of a few millimetres in diameter and transferred into 30 mL of 0.18% Protease Type XIV (Sigma-Adlrich, P5147) in Hank’s buffer (Thermo Fisher Science, 14175095). After dissociation for 2 h at 37 °C, cells were transferred to PureCol^®^(Advanced BioMatrix, 5005)-coated flasks (75 cm²) and cultured in Small Airway Epithelial Cell Growth medium supplemented with 1.0 µM A 83 − 01, 0.48 µM CHIR99021, 0.2 µM DMH-1 and 5 µM RI (ACDRI). After expansion, cells were dissociated with TrypLE™ (Thermo Fisher Science, 12604013) and seeded at a density of 1.77 × 10^5^ cells cm^− 2^ on ThinCert^®^ inserts or Costar^®^ Snapwell™ inserts coated with PureCol^®^. Cells were first cultured in Small Airway Epithelial Cell Growth medium supplemented with ACDRI for 48 h. Then, the media was changed to PneumaCult™-ALI medium. ALI cultures were provided with 0.5 mL of medium from the apical side and 1–3 mL from the basolateral side. ALI cultures were provided with 0.5 mL of medium from the apical side and 1–3 mL from the basolateral side. After 48 h the apical medium was removed to induce proper ALI cultivation. Following cells were matured and media changes were conducted every two to three days. Molecular and functional analyses were performed on day 37/38 of differentiation. For differentiation and analyses, all healthy (WT) donor-derived pALI culture were utilized in passage 3 and all CF patient (CF) donor-derived pALI cultures were utilized in passage 5. Before the analyses, all pALI cultures were treated with IL-13 (10 ng mL^− 1^) and partially ETI as previously described for iALI cultures. All cells were maintained at 37 °C and 5% CO_2_.

###  Immunofluorescence staining

iALI and pALI cultures were fixed with 0.5 mL 4% paraformaldehyde (Morphisto, 11762) at room temperature (RT). The insert membrane was utilized to perform staining of either the top viewed whole membrane or side viewed membrane sections. For section staining, the insert membrane was first paraffin-embedded, cut and then further processed for staining as described in *von Schledorn et al.* 2023 [69]. For whole membrane staining, permeabilization was performed with 0.5 mL TBS buffer with donkey serum (GeneTex, GTX73245) for 20 min at RT. The following primary antibodies were utilized and diluted in PBS plus 1% bovine serum albumin (BSA) (Sigma-Aldrich, A9418): MUC5AC (Thermo Fisher Science, MA5-12178, 1:200), TUBB4 (Cell Signaling Technology, 5335 S, 1:800). The following secondary antibodies were utilized and diluted in PBS plus 1% BSA: Cy™3 anti-rabbit IgG (Jackson ImmunoResearch, 711-165-152, 1:200), Alexa Fluor^®^ 488 anti-mouse IgG (Jackson ImmunoResearch, 715-545-151, 1:200). 0.5mL of primary antibodies were incubated over night at 4 °C. 0.5mL of secondary antibodies were incubated for 30 min at RT. Nuclei were stained with DAPI for 15 min at RT. For imaging the membrane was mounted on a glass slide in Fluorescence mounting medium (Agilent Dako, S3023). For membrane section staining, the membrane was first dehydrated and then embedded in paraffin. The membrane was cut into sections of 3 μm in thickness and transferred onto glass slides for staining. Primary and secondary antibodies as well as DAPI were applied as described above. Fluorescence imaging and image processing were performed with an AxioObserver A1 fluorescence microscope and AxioObserver Z1 fluorescence microscope and the ZENPro Sofware 3.0. For BSND staining samples were blocked and permeabilized using 0.25% (v/v) Triton-X 100 in PBS with 3% BSA for 60 min at RT, then incubated overnight at 4 °C with the anti-BSND antibody (Abcam, ab196017, 1:500) diluted in the Triton/BSA buffer. The samples were rinsed three times for 5 min with PBS before incubation with secondary antibody (Invitrogen, A11008, 1:500) diluted in Triton/BSA buffer for 1 h at 37 °C, followed by a triple 5 min wash with PBS. Then, samples were incubated for 1 h at 37 °C in Triton/BSA buffer containing directly conjugated anti- TUBB4 (Santa Cruz, sc-23950, 1:200) followed by a triple 5 min wash with PBS. The samples were stored at 4 °C until mounting. The samples were mounted by removing the cell culture membrane from the insert using a scalpel and placing the membrane onto a glass slide with the cells facing upwards. The cell was coated with a drop of ProLong™ Diamond Antifade Mountant (Invitrogen, P36965) and covered with a round number 1.5 glass coverslip.

### Composition analysis of ALI cultures via quantification of immunofluorescence signal area

Analysis was performed on ALI cultures after immunofluorescence staining of TUBB4 and MUC5AC, as earlier described. ImageJ/Fiji software was utilized to determine the area of TUBB4^+^ and MUC5AC^+^ signal after fluorescence microscopy. Input images were used in 16 bit file format. First, threshold was manually set using the ‘moments’ function. Afterwards signal-positive area was measured. Analysis was performed on multiple regions of interest (each 879µm x 879µm) per sample group.

### Flow cytometry analysis

Flow cytometry analysis was performed to quantify the expression of definitive endoderm and lung progenitor markers on day 3 and day 13 of hiPSCs differentiation into respiratory epithelial cells, respectively. On day 3, live cell staining was performed. On day 13, cells were fixed and permeabilized using the FoxP3 staining buffer set (Miltenyi Biotec, 130-093-142). Each 10^5^ cells were taken and suspended in 100 µL PBS with 1% BSA. The following directly-labelled primary antibodies were utilized: APC anti-CXCR4 (Thermo Fisher Science; 17-9999-42, 1:25), APC anti-NXK2.1 (Miltenyi Biotec, 130-118-309, 1:2,000), PE anti-c-Kit (Thermo Fisher Science, 12-1178-42, 1:33), PE anti-EpCAM (BD Biosciences, 347198, 1:33). Primary antibodies were diluted in 1% BSA (Sigma-Aldrich, A9418) in PBS w/o (Thermo Fisher Science, 70011044) and incubated for 30 min on ice. Flow cytometry analysis was performed with a MACSQuant Analyzer 10 and FlowJo analysis software.

### Real-time quantitative PCR (RT-qPCR)

RNA samples from cells were collected in Trizol^®^ reagent (Invitrogen, 15596018) RNA isolation was performed using the NucleoSpin^®^ RNA II kit (Macherey-Nagel, 740955.50) and cDNA synthesis was performed using the RevertAid™ H Minus First Strand cDNA Synthesis kit (Thermo Fisher Science, K1631). Real-time qPCR analysis was carried out using the SsoAdvanced™ Universal SYBR^®^ Green Supermix (Bio-Rad Laboratories, 1725270) and CTX Connect Real-Time PCR Detection system (Bio-Rad Laboratories). Target gene expression was normalized to the expression of house-keeping genes bACT and GAPDH. Applied primer pairs and sequences are listed in supplemental data set.

###  Western blot analysis

Western blot analyses were performed as previously described [56]. The following antibodies were utilized: Beta-actin (Abcam, ab8226), CFTR (Cystic Fibrosis Foundation CFTR Antibody Distribution Program, Chapel Hill, North Carolina; antibody mix: 596 + 570 + 217 + 660), TMEM16A (Abcam, ab64085); ZO-1 (Invitrogen, 33-9100). 16HBE14o- cells (human bronchial epithelial cells; HBE) were utilized as a control for protein expression in airway epithelial cells.

###  Measurement of the transepithelial ion conductance

Recordings of the transepithelial ion conductance in iALI and pALI cultures was performed in EasyMount Ussing chambers (Physiologic Instruments) using voltage clamp configuration to measure the transepithelial short-circuit current (*I*_SC_). The *I*_SC_ was continuously recorded using Lab-Chart8 (AD Instruments), and transepithelial resistance (*R*_TE_) was monitored by application of short voltage pulses (1 mV) every 60 s. Experiments were performed in Ringer buffer as previously described [87, 88]. After 10–15 min equilibration, basal *I*_SC_ was measured and amiloride (100 µM; Sigma-Aldrich, A7410) was added to inhibit sodium absorption via ENaC. Next, forskolin (10 µM; Sigma-Aldrich, F6886) and 3-isobutyl-1-methylxanthin (IBMX; 100 µM; Sigma-Aldrich, I5879) were added together, followed by CFTR-inhibitor 172 (20 µM; CFTRinh-172) (TargetMol, T2355) to assess CFTR-mediated chloride conductance. Uridine-triphosphate (UTP; 10 µM; Thermo Fisher Science, R1471) was added to evaluate the calcium-activated chloride conductance. Both, the activation of CFTR by forskolin and IBMX as well as the activation of CaCCs were measured as the peak response after compound addition. Lastly, GlyH-101 (50 µM; AbMole, M6754) and subsequently niflumic acid (NFA; 500 µM; Cayman Chemical Company, 70650) were applied to inhibited residual anion conductance.

###  Measurement of the ciliary beat frequency (CBF)

Measurement of the *CBF* on iALI and pALI cultures was performed via high-frequency video microscopy imaging using a Zeiss Axiovert A.1 microscope equipped with a Basler SCA640 120FM camera in a humidified environmental chamber at 37 °C and in 5% CO2. Videos were recorded with a 40x magnification objective at 100 frames per second using a phase contrast filter. Videos were recorded in at least 15 positions per insert, selected in a meandering pattern throughout the whole insert, avoiding the edges with the meniscus. *CBF* and cilia coverage were analyzed using Sisson-Ammons video analysis (SAVA) software [89], where average *CBF* and area of moving pixels were measured by whole field analysis. Inserts with less than 10,000 moving pixels (< 0.01%) of total imaged area were determined as non-motile. Data are show as average per insert. Apical washes were performed with PBS 24 h before the measurement.

### Transmission electron microscopy

ALI cultures were fixed in 150 mM HEPES buffer (pH 7.35) containing 1.5% glutaraldehyde and formaldehyde at RT for 20 min and at 4 °C over night. 1.5 mm sized pieces of the fixed cultures were high pressure frozen in a HPM 100 (Leica Microsystems, Wetzlar). Freeze substitution was carried out in a Leica AFS (Leica Microsystems, Wetzlar) in acetone containing 0,1% tannic acid at -90 °C over night and after washing in acetone continued in acetone containing 2% osmiumtetroxide. Temperature was raised to -20 °C and after 2 h to 4 °C. After washing in acetone, samples were transferred to RT and embedded in EPON. 50 nm thick cross-sections of the ALI-cultures were poststained with uranyl acetate and lead citrate (Reynolds et al. 1963) and observed in a Zeiss EM 900 (Zeiss, Oberkochen), operated in the bright field mode at 80 kV. Images were recorded with a side-mounted 4k CCD-camera (TRS, Dünzelbach). For estimation of the mucus layer height, complete 1.5 mm profiles were recorded iteratively and the mucus layer was estimated every 6 μm at 90 degree to the median cell surface on the respective images.

### Scanning electron microscopy

After fixation as for TEM, ALI cultures were washed in water, critical point dried and sputtered with gold. Examination was done in a Crossbeam 540 (Zeiss, Oberkochen) at 10 kV.

### Statistical analyses

GraphPad Prism6 was utilized to perform statistical analyses. Results are presented as means ± SD unless otherwise noted. For analysis a one-way ANOVA and post hoc Tukey test were performed. In regards to all functional measurements, no comparisons between the iALI and pALI test group were made due to difference in purity and presumably maturity of the cells.

### Use of Artificial Intelligence

For the purpose of supporting the writing process of this manuscript, Meta LLaMA 3.1 8B Instruct and OpenAI ChatGPT-4 and DeepL were sporadically used to rephrase individual sentences.

## Supplementary Information

Below is the link to the electronic supplementary material.

Supplementary Material 1

Supplementary Material 2

Supplementary Material 3

## Data Availability

The underlying data to all graphs is available in the supplementary data sheets Supplementary Table 1. Supplementary material information are available in Supplementary Table 2.

## Abbreviations

ALI: Air-liquid-interface
BSND: Barttin CLCNK Type Accessory Subunit Beta
CaCCs: Calcium-activated chloride channel
cAMP: Cyclic adenosine monophosphate
CBF: Ciliary beat frequency
CF: Cystic fibrosis
CFTR: Cystic fibrosis transmembrane conductance regulator
CXCR4: C-X-C chemokine receptor type 4
DE: Definitive endoderm
ENaC: Epithelial sodium channel
EpCAM: Epithelial cell adhesion molecule
ETI: Elexacaftor-tezacaftor-ivacaftor
FIS: Forskolin-induced swelling
hiPSC: Human induced pluripotent stem cell
iALI culture: hiPSC-derived ALI culture
I_SC_: Short-circuit current
LP: Lung progenitor
MACS: Magnetic-activated cell sorting
MCC: Mucociliary clearance
MLH: Mucus layer height
MUC5AC: Mucin 5AC
n: Replicate
NKX2.1: NK2 homeobox 1
ns: None significant
pALI culture: primary ALI culture
SCGB1A1: Secretoglobin family 1 A member 1
SD: Standard deviation
SEM: Scanning electron microscopy
SLC26A9: Solute carrier family 26 member 9
TEM: Transmission electron microscopy
TMEM16A: Transmembrane member 16 A
TUBB4: Acetylated tubulin beta-4 chain
WT: Wild type (healthy)
