# Supplementary material for: Human induced pluripotent stem cells for in vitro modeling of impaired mucociliary clearance in cystic fibrosis lung disease
Source: Stem Cell Res Ther. 2025 Oct 21;16:573. doi: 10.1186/s13287-025-04737-0 (PMC12538740; doi:10.1186/s13287-025-04737-0)
Supplement: Supplementary file 3 — Supplementary Material 3 [file 13287_2025_4737_MOESM3_ESM.docx]

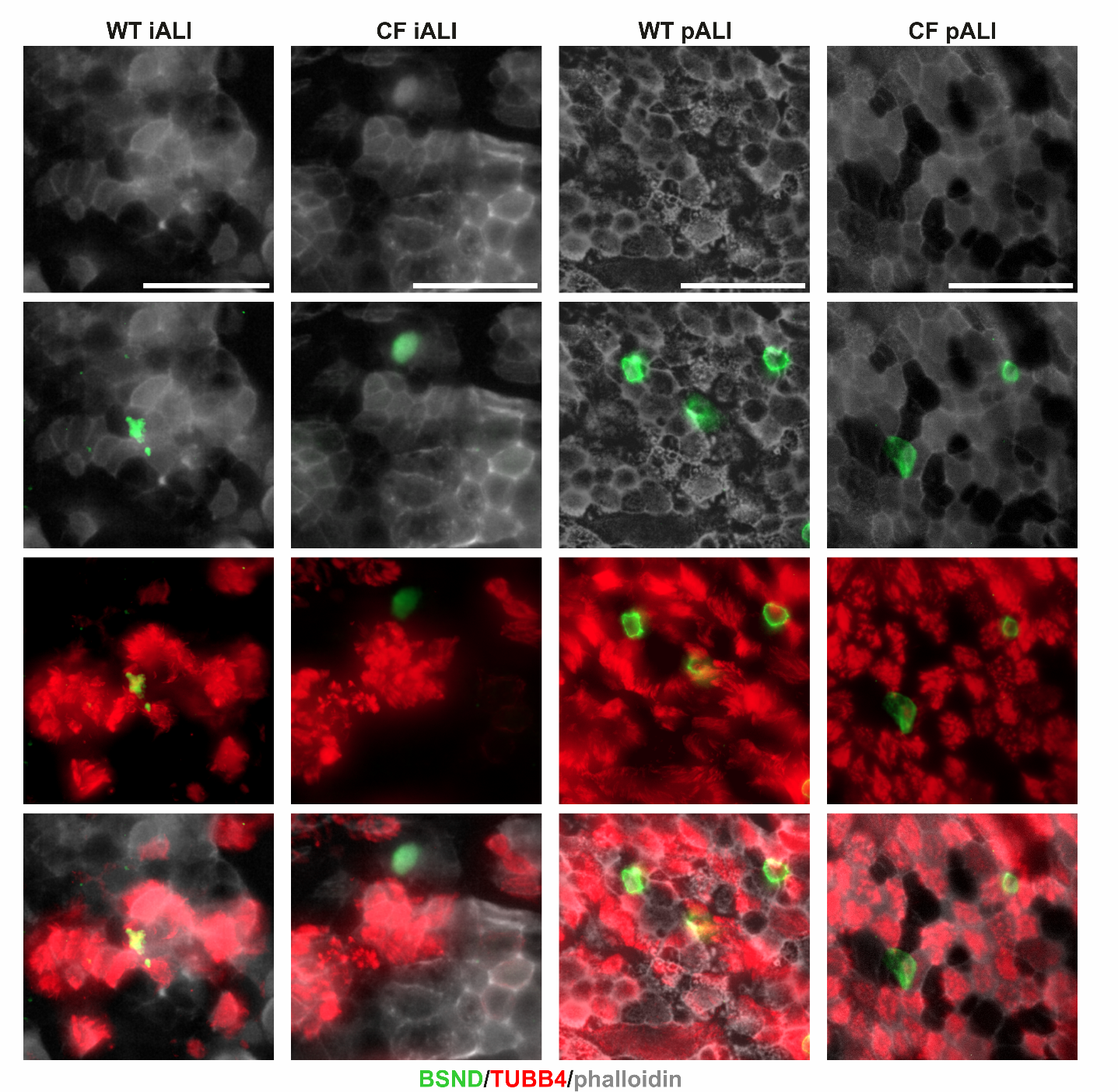


Supplemental Figure 1: Immunofluorescence staining demonstrate the presence of BSND^+^ ionocytes and TUBB4^+^ ciliated cells in iALI and pALI cultures.

Whole mount immunofluorescence staining of ALI insert membranes showing the expression of BSND^+^ ionocytes, TUBB4^+^ ciliated cells and phalloidin (scale bar: 40 µm).


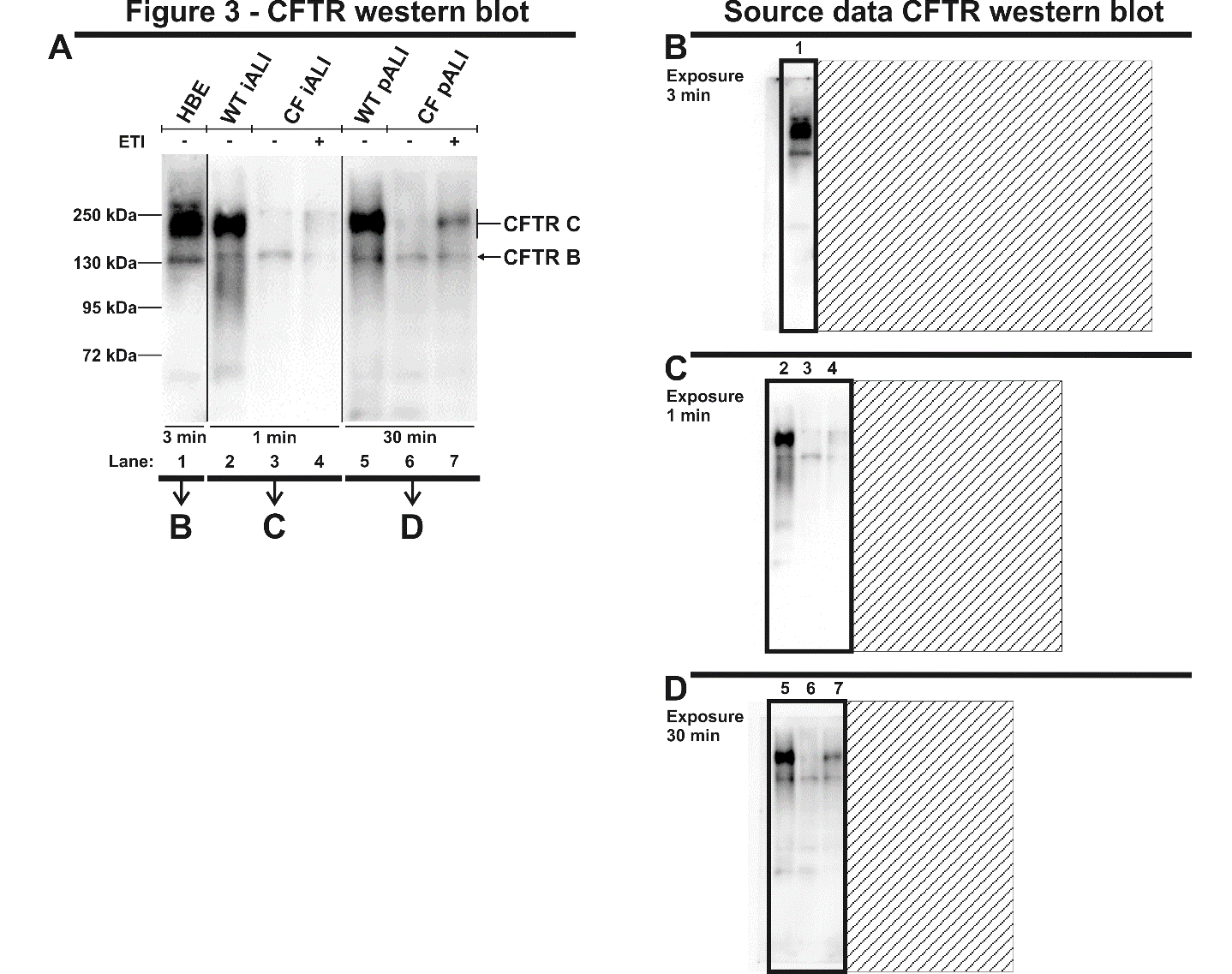


Supplemental Figure 2: Source data for Figure 3 – CFTR western blot analysis of iALI and pALI cultures.

Figure 4 is composed of three different western blot membranes showing representative results of the analysis. Western blot analyses were performed on iALI and pALI cultures as well as human bronchial epithelial cells (HBE) as a control. Treatment with CFTR modulators ETI was applied for 48 h (WT – Healthy, CF – homozygous CFTR Phe508del, - vehicle, + ETI-treated). (A) CFTR western blot results shown in Figure 4. (B-D) Western blot membranes showing the complete lanes that were used for the generation of Figure 4. Presented exposure times were selected for optimal display of banding pattern and are highlighted below the blot lanes.


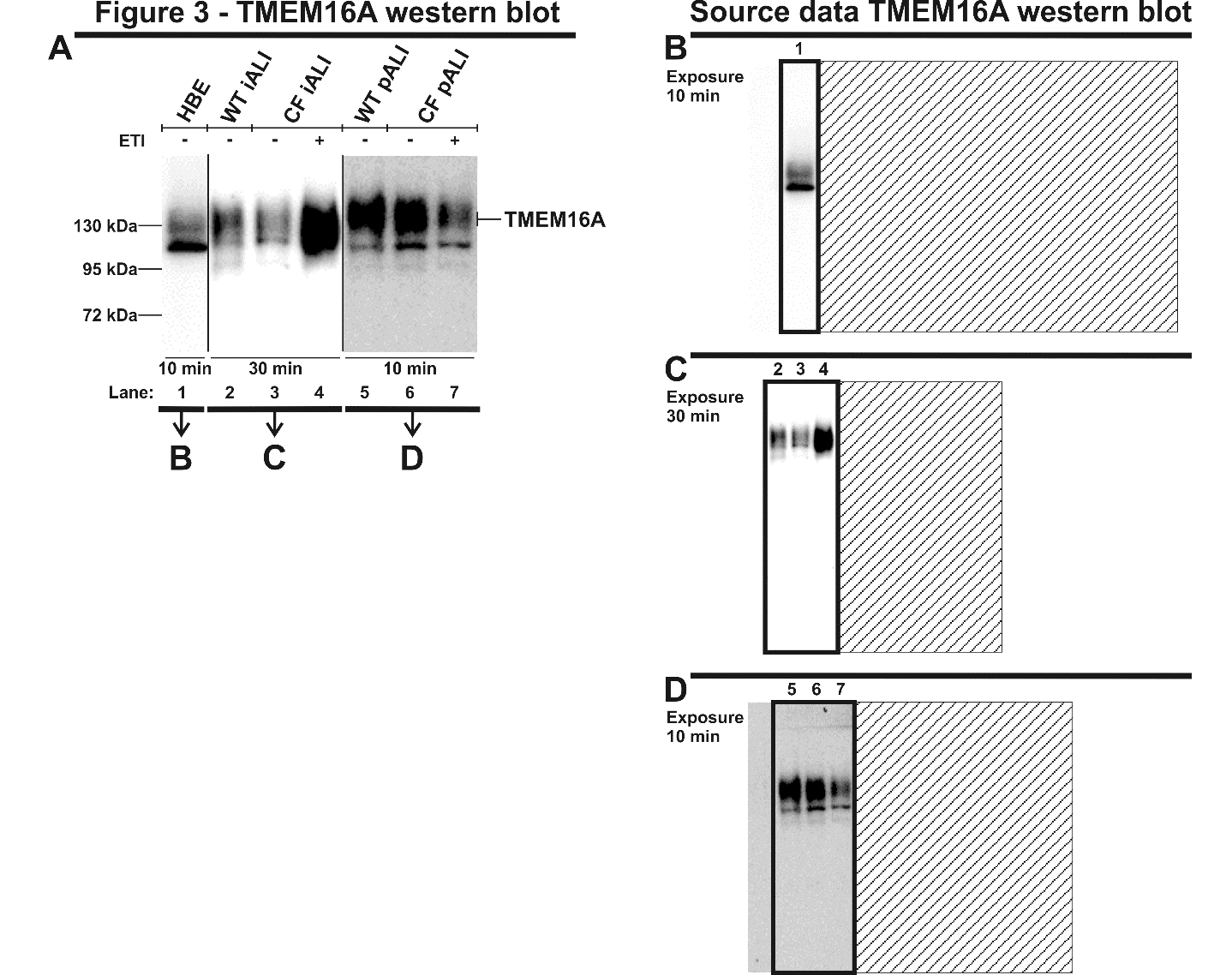


Supplemental Figure 3: Source data for Figure 3 – TMEM16A western blot analysis of iALI and pALI cultures.

Figure 4 is composed of three different western blot membranes showing representative results of the analysis. Western blot analyses were performed on iALI and pALI cultures as well as human bronchial epithelial cells (HBE) as a control. Treatment with CFTR modulators ETI was applied for 48 h (WT – Healthy, CF – homozygous CFTR Phe508del, - vehicle, + ETI-treated). (A) TMEM16A western blot results shown in Figure 4. (B-D) Western blot membranes showing the complete lanes that were used for the generation of Figure 4. Presented exposure times were selected for optimal display of banding pattern and are highlighted below the blot lanes.


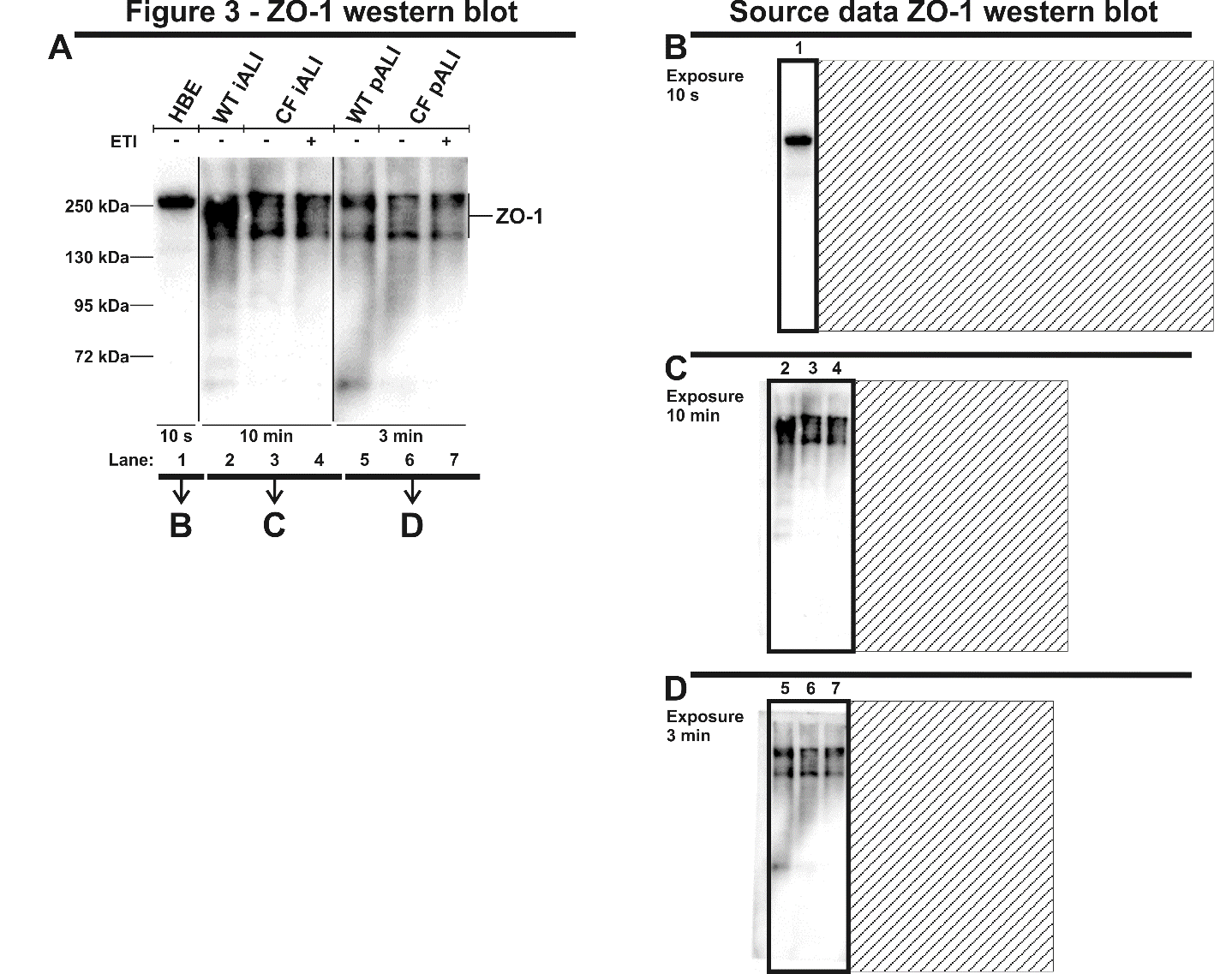


Supplemental Figure 4: Source data for Figure 3 – ZO-1 western blot analysis of iALI and pALI cultures.

Figure 4 is composed of three different western blot membranes showing representative results of the analysis. Western blot analyses were performed on iALI and pALI cultures as well as human bronchial epithelial cells (HBE) as a control. Treatment with CFTR modulators ETI was applied for 48 h (WT – Healthy, CF – homozygous CFTR Phe508del, - vehicle, + ETI-treated). (A) ZO-1 western blot results shown in Figure 4. (B-D) Western blot membranes showing the complete lanes that were used for the generation of Figure 4. Presented exposure times were selected for optimal display of banding pattern and are highlighted below the blot lanes.


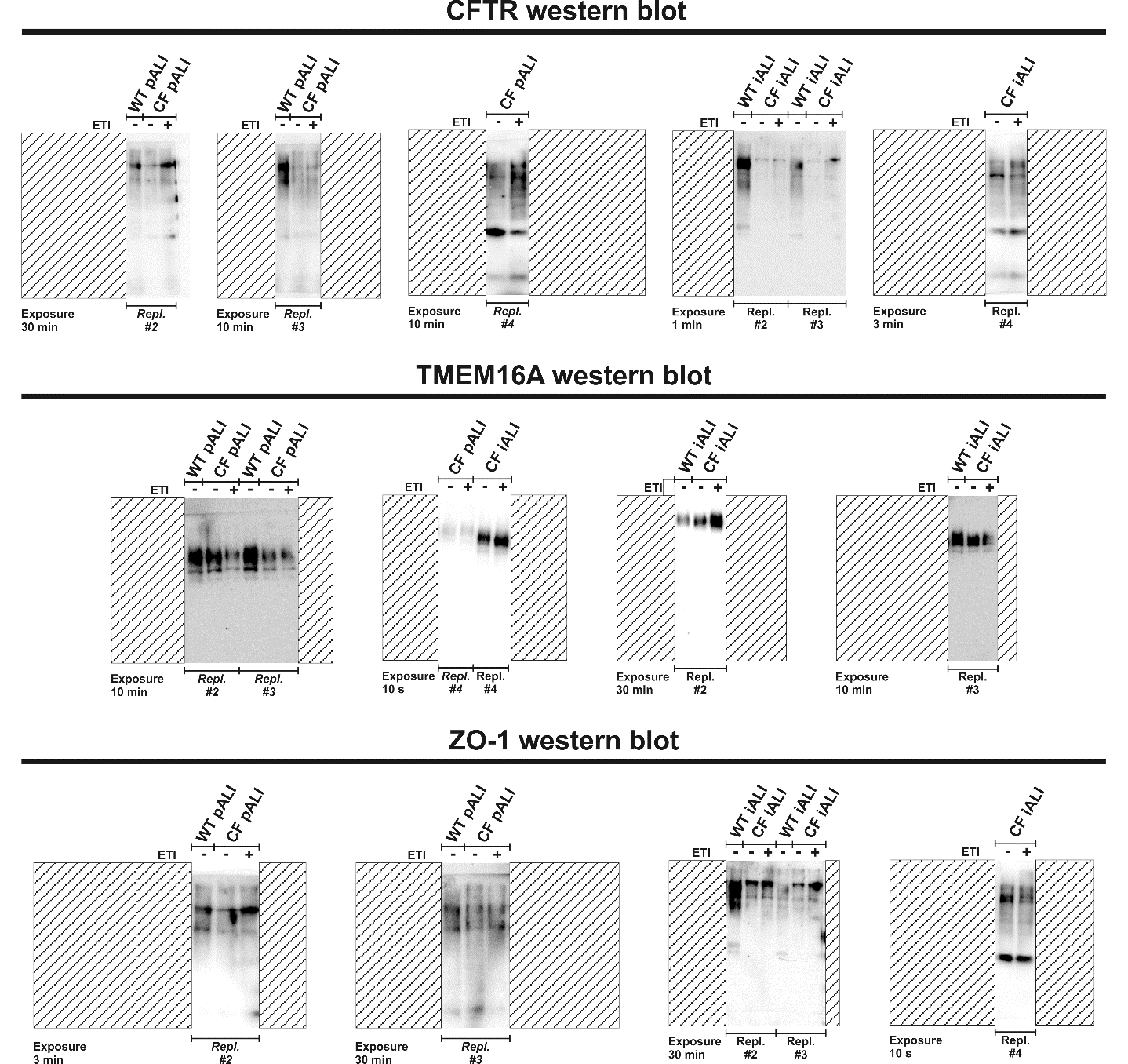


Supplemental Figure 5: Source data showing the additional biological replicates of the CFTR, TMEM16A and ZO-1 western blot analyses of iALI and pALI cultures.

CFTR, TMEM16A and ZO-1 western blot analyses were performed as biological replicates. Replicates represent number of ALI cultures derived from independent differentiations: n=3-4 per group. Western blot analyses were performed on iALI and pALI cultures as well as human bronchial epithelial cells (HBE) as a control. Treatment with CFTR modulators ETI was applied for 48 h (WT – Healthy, CF – homozygous CFTR Phe508del, - vehicle, + ETI-treated). For each experimental group, replicate #1 is shown in Figure 4. The additional replicates, #2-4, are presented in this supplemental figure. Presented exposure times were selected for optimal display of banding pattern and are highlighted below the blot lanes.


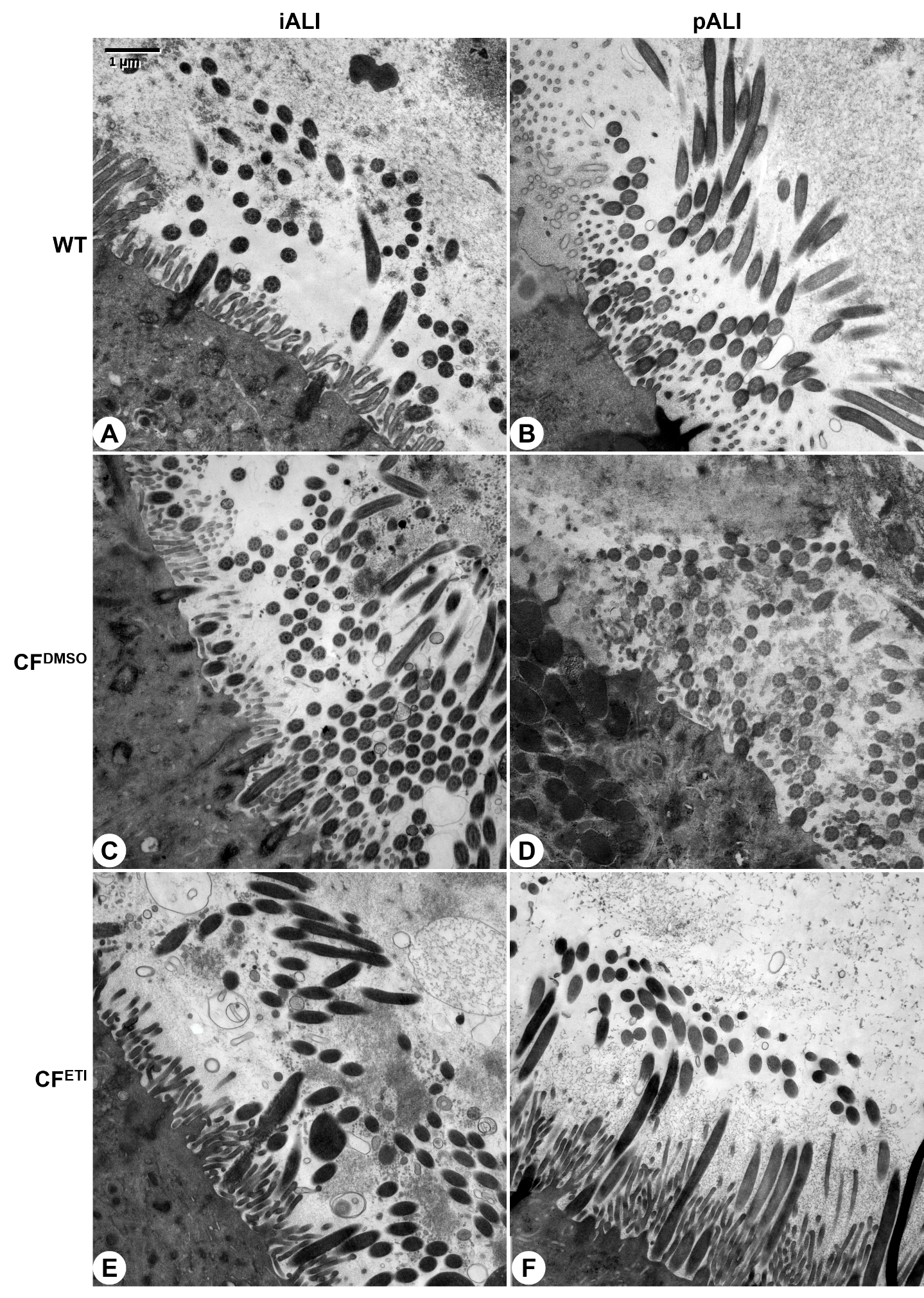


Supplemental Figure 6: Transmitted electron microscopy confirms integrity of the cilia on iALI and pALI cultures.

Transmitted electron microscopy was performed on (A+C+E) iALI and (B+D+F) pALI cultures. (A+B): WT; (C+D): CF^DMSO^; (E+F): CF^ETI^. Treatment with CFTR modulators ETI was applied for 24 h (WT – Healthy, CF – homozygous CFTR ΔF508, CF^DMSO^ – vehicle, CF^ETI^ – ETI-treated). Results are shown at medium magnification to view cilia and microvilli. Scale bar shown in (A). Analysis was performed on one biological replicate per group (n=1).
